# The Chromatin Structure at the *MECP2* Gene and In Silico Prediction of Potential Coding and Non-Coding *MECP2* Splice Variants

**DOI:** 10.3390/ijms232415643

**Published:** 2022-12-09

**Authors:** Danilo Shevkoplyas, Yen My Vuu, James R. Davie, Mojgan Rastegar

**Affiliations:** Department of Biochemistry and Medical Genetics, Max Rady College of Medicine, Rady Faculty of Health Sciences, University of Manitoba, Winnipeg, MB R3E 0J9, Canada

**Keywords:** epigenetics, MeCP2 isoforms, alternative splicing, genomics, RTT, UCSC, ATAC-seq

## Abstract

Methyl CpG binding protein 2 (MeCP2) is an epigenetic reader that binds to methylated CpG dinucleotides and regulates gene transcription. *Mecp2*/*MECP2* gene has 4 exons, encoding for protein isoforms MeCP2E1 and MeCP2E2. MeCP2 plays key roles in neurodevelopment, therefore, its gain- and loss-of-function mutations lead to neurodevelopmental disorders including Rett Syndrome. Here, we describe the structure, functional domains, and evidence support for potential additional alternatively spliced *MECP2* transcripts and protein isoforms. We conclude that NCBI MeCP2 isoforms 3 and 4 contain certain MeCP2 functional domains. Our in silico analysis led to identification of histone modification and accessibility profiles at the *MECP2* gene and its *cis*-regulatory elements. We conclude that the human *MECP2* gene associated histone post-translational modifications exhibit high similarity between males and females. Between brain regions, histone modifications were found to be less conserved and enriched within larger genomic segments named as “S1–S11”. We also identified highly conserved DNA accessibility regions in different tissues and brain regions, named as “A1–A9” and “B1–B9”. DNA methylation profile was similar between mid-frontal gyrus of donors 35 days–25 years of age. Based on ATAC-seq data, the identified hypomethylated regions “H1–H8” intersected with most regions of the accessible chromatin (A regions).

## 1. Introduction

Gene regulation is a complex process, controlled at multiple different layers including: the pre-, post-, and/or intra-transcriptional levels, followed by several translational and pos-translational regulation steps. These processes would help individual eukaryotic cells to control and determine their number of transcripts and protein levels [1,2,3].

Gene transcription is mainly controlled by corresponding *cis*-regulatory elements (*cis*-REs) and *trans*-acting transcription factors [3]. In general, *cis*-regulatory elements are considered as DNA binding sites for *trans*-acting factors. When associated, these can initiate, regulate, and terminate gene transcription. *Cis*-regulatory elements include promoters (core, proximal, and distal), enhancers, silencers, and insulators. The core promoters encompass the TATA box, B recognition element (BRE), downstream promoter element (DPE) and Initiator element (Inr). These sequences are located relative to the transcription start site (TSS). Core promoter elements consist of essential binding sites for RNA transcription machinery, and act in an orientation-dependent manner [4,5]. Enhancers stimulate gene expression and can be located close or far from the gene, and act in an orientation- independent manner, while silencers act as suppressors of gene expression. Insulator elements prevent improper interactions between enhancers or silencers and other non-target genes [6]. Proximal promoter is located upstream of the core promoter and may function as the tethering sequence that assists in the interaction between enhancers and core promoter elements [4,7], while distal promoters’ location can be more than 1 kilobase (kb) upstream of the TSS.

Epigenetic control refers to gene regulatory mechanisms that do not directly involve DNA nucleotide sequences within a gene, and may include conformational change in the chromatin structure [8]. Epigenetic modifications include different forms of DNA methylations (such as 5-methyl cytosine [5-mC] and 5-hydroxy-methyl cytosine [5-hmC]) as well as methylation, acetylation, ubiquitination, SUMOylation, and phosphorylation of histones [9]. The high abundance of 5-mC in the promoters is generally associated with transcriptional inhibition through: (a) preventing the transcription activator binding, (b) recruitment of co-repressive complexes, and the (c) crosstalk with post-transcriptional modifications. Unlike 5-mC, 5-hmC abundance is mainly associated with active chromatin conformational state. In addition to gene regulation, DNA methylation is also important for X-chromosome inactivation, genome organization, imprinting, and RNA splicing [9].

Epigenetic factors include epigenetic “writers”, “erasers”, and “readers” [3,10]. Epigenetic writers are enzymes that can add certain functional groups to DNA nucleotides or histone proteins, while erasers are enzymes that remove or alter these modifications. Epigenetic readers recognize these DNA or histone modifications and may employ additional factors to regulate gene transcription [11,12]. 

Methyl CpG binding protein 2 (MeCP2) is a nuclear epigenetic reader that can bind to methyl-CpG dinucleotides and employ other proteins to alter the activity of gene transcription [3]. In humans, MeCP2 protein is encoded by the *MECP2* gene located on the X-q28 (National Center for Biotechnology Information [NCBI], Gene 4204), while in mice (*Mus musculus*) it is produced from the *Mecp2* gene located on the X A7.3 (NCBI, Gene 17257). The *MECP2*/*Mecp2* gene has 4 exons, currently known to encode for two main protein isoforms, MeCP2E1 (E1) and MeCP2E2 (E2), produced by alternative splicing. MeCP2E1 (also known as MeCP2α or M2CP2B) is translated from a transcript variant, which includes exons 1, 3, and 4, with a translation start site within the exon 1. MeCP2E2 (also known as MeCP2β or MeCP2A) is translated from a transcript variant, that includes exons 1–4 (and mature transcript with exons 2, 3, 4) and a translation start site located in exon 2 [13]. In addition to these two alternative splicing isoforms, several different 3′UTR isoforms result from alternative polyadenylation both in mice and humans [14]. 

MeCP2 was first discovered in rats by Dr. Adrian Bird and his team as a protein that can sufficiently bind to a single methyl-CpG site [15], whereas previously discovered MeCP1 required over 10 methylated CpG nucleotide pairs to efficiently bind to DNA [16]. It was later identified that MeCP2 has an equally high affinity for both 5-mC and 5-hmC [17]. MeCP2 can also bind to unmethylated DNA [18], and condensed chromatin by interacting with the linker DNA between nucleosomes [19]. When bound to DNA, MeCP2 recruits other factors including mSin3a co-repressor, histone deacetylases HDAC1 and HDAC2 [20], and histone methyltransferases [21], to condense the chromatin. Other MeCP2 co-repressors include SMRT [22], N-CoR [23], and ATRX [24]. MeCP2 may also interact with TFIIB to negatively regulate gene transcription [25], while impacting global transcription [26], and protein translation [26,27], with additional links to fundamental cell signaling pathways [27,28]. In addition to acting as a negative regulator of gene transcription, MeCP2 also acts as a transcriptional gene activator reported in mice hypothalamus [29]. This suggests that loss-of-function mutations in the *MECP2/Mecp2* gene may result in both up-regulation of some genes and downregulation of others, and consequently cellular dysfunction, where MeCP2 is most abundant.

MeCP2 relative abundance is highest in the brain (especially in mature neurons), lungs, and spleen [30], with MeCP2 levels being controlled by tissue- and cell type-specific regulatory mechanisms [30,31,32,33,34,35]. Brain region- and cell type-specific expression of MeCP2 isoforms correlate with DNA methylation of CpG sites within *cis*-regulatory elements in the mouse *Mecp2* promoter and the first intron [31,32,35,36]. It has been suggested that MeCP2 homeostasis regulation in the brain is region-dependent and might be based on the *MECP2-BDNF-miR132* feedback regulatory loop [37], although correlational studies in humans do not support such a feedback regulatory loop [38]. In mouse and human brains, the timing of MeCP2 expression correlates with the development of the central neural system with preliminary expression in the brainstem and spinal cord, and later expression in the cerebral cortex and hippocampus [30]. MeCP2 expression in the cerebral cortex has also been shown to associate with the maturation of neurons [30]. Isoform-specific expression analysis of primary murine brain cells showed that in male neurons, *Mecp2e1* is the major isoform with about 3-fold higher levels than *Mecp2e2* [34]. MeCP2 expression throughout development is different between mice and humans. In humans, the increase in percentage of MeCP2 positive neurons is observed up to 10 years of age with the highest increase during gestation. While in mice, such increase was confined to the embryogenesis stage with the maximum number of MeCP2 expression in neurons by E18.5 (embryonic stage). The reason for this might be a longer period of developmental plasticity in humans, suggesting that MeCP2 might alter the expression of the genes important for neuronal development [30,39]. *MECP2* is regulated both transcriptionally and post-transcriptionally [27,37,40]. 

As MeCP2 is highly involved in neurodevelopment, either dysfunction or increased dose of MeCP2 protein levels caused by genetic mutations may lead to a variety of neural pathological abnormalities. Rett Syndrome (RTT) is caused by missense, nonsense, and/or frameshift loss-of-function mutations in the *MECP2* gene [37,41,42]. RTT is a neurodevelopmental disease occurring almost exclusively in females, although rare cases of male patients are also reported even until adulthood [37,43,44]. Rett Syndrome is characterized by initial normal development followed by stagnation and deterioration of brain functions between 6 to 18 months of development. By the age of 1.5 years, RTT patients experience autism, dementia, truncal ataxia, acquired microencephaly and loss of purposeful use of hands and verbal communication ability [45]. *MECP2* duplications cause *MECP2* Duplication Syndrome in males. The symptoms include hypotonia, mental disability, mild dysmorphic features such as depressed nasal bridge, upturned nares, large ears, ataxia, and autistic features [46]. This suggests that MeCP2 must be appropriately balanced throughout development.

As indicated, the two known MeCP2 isoforms (E1 and E2) are produced by alternative splicing. Additional potential isoforms of MeCP2 have not been fully explored, even though large data depositories such as NCBI, Ensembl, and UniProt list more than two potential protein-coding *MECP2* transcript variants. 

The National Centre of Biotechnology (NCBI) is part of the National Library of Medicine. It is a large data depository composed of interconnected sub-databases dedicated to genes, gene products, homology, and taxonomy, among others. NCBI offers a wide variety of automated algorithms and pipelines to make data annotation autonomous which makes it possible to update the database daily. NCBI Reference Sequence (RefSeq) database is a project curated by NCBI staff aiming to provide a frequently updated, non-redundant, and thoroughly annotated set of reference standards which are obtained from the International Nucleotide Sequence Database Collaboration (INSDC). The main difference between INSDC and NCBI RefSeq is that while INSDC is an archival repository containing many versions of the same sequences, NCBI RefSeq consists of the most recently updated chromosomal, genomic, and protein sequences [47]. In RefSeq, all sequences can be divided into the model- and known sequences. Model sequences are generated from the eukaryotic genome annotation pipeline and have the following accession number styles: XM_, XR_, XP_ (underscore in the accession number defines sequence as a part of RefSeq). Known sequences are derived from GenBank EST and cDNA data and then are reviewed by NCBI members; such sequences use the following accession number styles: NM_, NR_, and NP_ [47]. All RefSeq records contain universal data evidence code and link to original RNA-seq data that was used to derive a given sequence. 

Ensembl is another project targeted at the automatization of genome annotation. The main goal of Ensembl has been the integration of annotation with other available data and making it accessible to the public. Regarding the annotation workflow, Ensembl is very similar to NCBI RefSeq. The sequencing data are automatically processed from large data archive repositories such as INSDC, dbSNP, GTEx, and Roadmap Epigenomics and then it is reviewed by their staff [48]. 

The Universal Protein Resource (UniProt) is a database composed of UniProt Reference Clusters (UniRef), UniProt Archive (UniParc), and UniProt Knowledgebase (UniProtKB). UniProt is based on the collaboration between the European Bioinformatics Institute (EMBL-EBI), Protein Information Institute (PIR), and the Swiss Institute of Bioinformatics (SIB). UniProtKB is further subdivided into Swiss-Prot and TrEMBL. TrEMBL (Translated EMBL Nucleotide Sequence Data Library) is composed of automatically annotated unreviewed sequences from EMBL, GenBank, DDBJ, Ensembl, etc., while SwissProt contains reviewed sequences from TrEMBL that have passed expert manual annotation [49]. 

In addition to in vitro or in vivo research, bioinformatic approaches can be used to filter, organize, and visualize publicly available whole-genome or epigenome sequencing data when such techniques are not available. Database portals such as ENCODE (www.encodeproject.org) and NCBI (www.ncbi.nlm.nih.gov) contain sub-databases dedicated to raw data from numerous types of publicly available sequencing experiments. Raw sequencing data files are provided in the format that specifies some value to a region or nucleotide position on a certain version of the organism genome. One of the easiest ways to visualize such data is to use pc- or online-based genome browsers such as UCSC Genome Viewing Browser [50] or IGV [51]. Genome viewing browsers read the raw format files and automatically overlay the values from the file onto the genome based on the nucleotide positions in each chromosome. Additionally, genome viewing browsers allow visualizing multiple different data files for a given chromosomal region, thus making it possible for a researcher to correlate the values and make predictions that might be relevant to their wet laboratory research. Genome viewing browsers do not require any advanced knowledge in bioinformatics or any programming language, as they provide a user-friendly and intuitive interface.

Despite the importance of MeCP2 in health and disease, our knowledge about *MECP2* coding and non-coding transcripts and protein isoforms is currently inconclusive. The objectives of this study were to analyze the potential MeCP2 protein isoforms using collection and integration of data from multiple databases. We also aimed to investigate *MECP2 cis*-regulatory elements by using whole genome sequencing data to assess the availability of chromatin structure around and within the *MECP2* gene.

## 2. Results

### 2.1. MECP2 Transcripts and Encoded Protein Isoforms

To study potential *MECP2* transcript variants, we first examined data from NCBI records via a summary of the extracted information presented in Table 1. NCBI records for the human *MECP2* gene (Gene ID: 4204) listed reviewed variants from RefSeq database (NM_). All NM_ variants listed in NCBI were declared to be protein-coding sequences and have respective protein isoforms.

**Figure 1:**
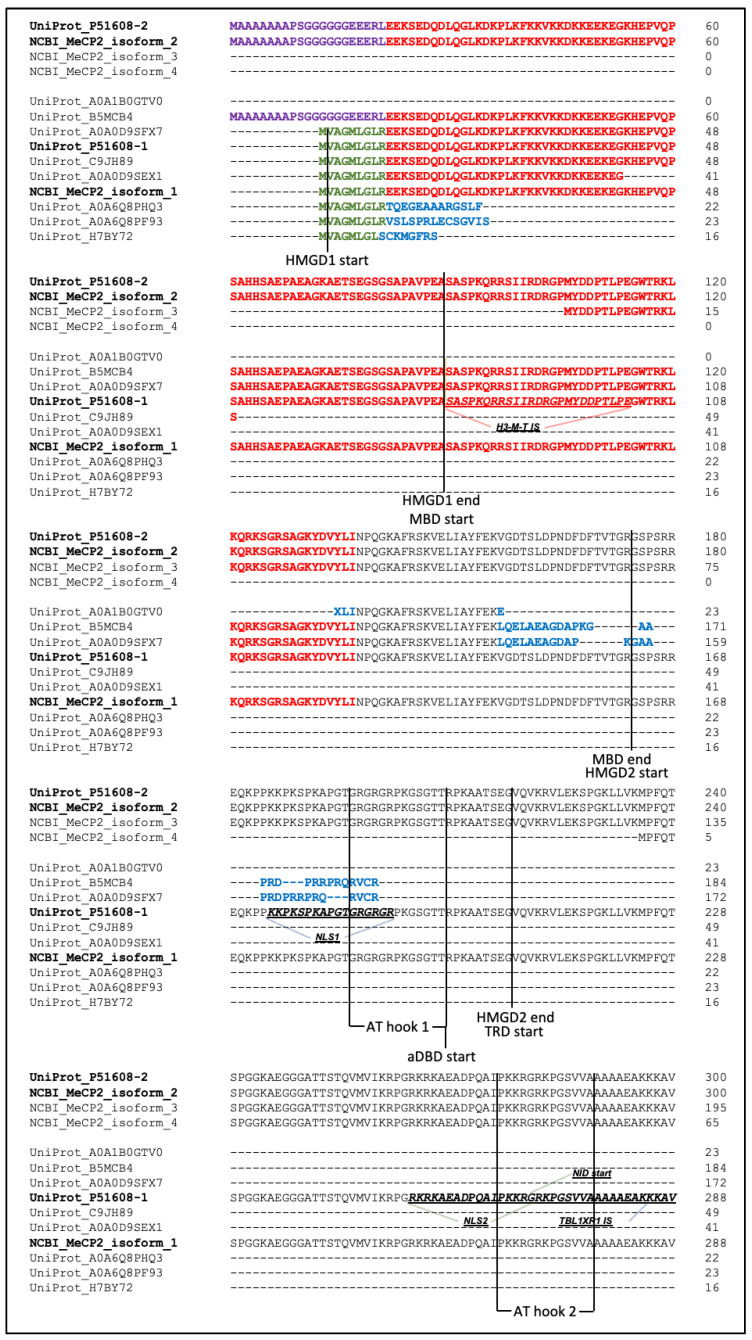

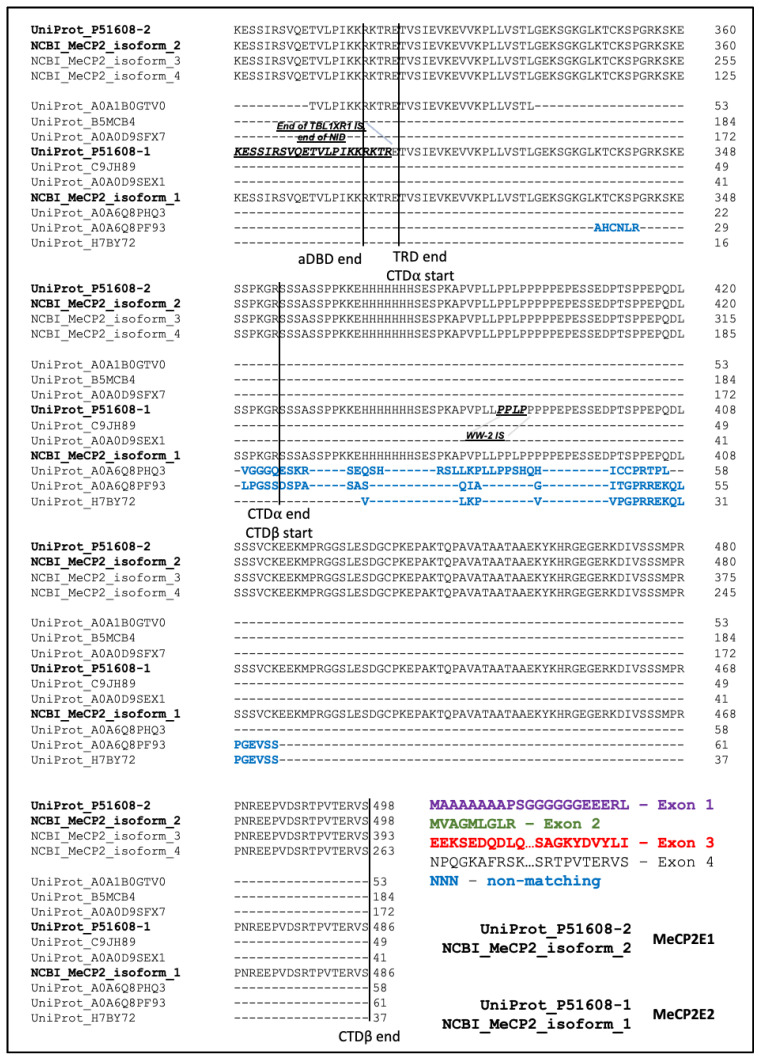
Alignment of potential MeCP2 protein isoforms from NCBI and UniProt. The sequences are in the N-terminal to C-terminal orientation. MeCP2E1 and MeCP2E2 from both NCBI and UniProt are shown in bold. The raw output from ClustalOmega was split into 2 panes and exons were colour-coded as indicated in the figure legend. Functional domains are labeled with start and end flags on MeCP2E2 (NCBI isoform 1 and UniProt P51608-1), while protein interaction sequences were underlined. The sequences of exons 1, 2, 3, and 4 were coloured purple, green, red and black respectively. Non-matching to either isoform sequences were coloured blue. aDBD: alternative DNA binding domain; CTDα: C-terminal domain α; CTDβ: C-terminal domain β; HMGD1: high mobility group protein-like domain 1; HMGD2: high mobility group protein-like domain 2; MBD: methyl-CpG-binding domain; NID: NCoR/SMRT interaction domain; NLS1: nuclear localization signals 1; NLS2: nuclear localization signals 2; TBL1XR1 IS: TBL1XR1 interaction sequence; TRD: transcriptional repression domain; WW-2 IS: Group 2 WW motif-containing protein interaction sequence. References and amino acid numbers of each feature were summarized in Supplementary Table S1.

The transcript variant 1 contains all 4 exons and translates to protein isoform 1 (MeCP2A or MeCP2E2). Protein translation from this transcript starts in exon 2 at a “dominant” downstream to exon 1 start codon which results in exon 1 exclusion from the protein. The transcript variant 2 results from an alternative splicing event of pre-mRNA, which excludes exon 2. Protein translation starts at the upstream start codon in exon 1 resulting in protein isoform 2 (MeCP2B or MeCP2E1). According to sequence alignment (Figure 1), these two isoforms differ in their N-termini. Isoform 2 (MeCP2E1) has 21 amino acids whereas isoform 1 (MeCP2E2) has 9 distinctive amino acids. Other than that, the two protein isoforms 1 and 2 are identical. These two isoforms are well described in the literature due to their relevance to neurodevelopmental disorders [13,33,36,38,52,53,54]. 

NCBI also lists eight more transcript variants potentially translating to two more protein isoforms. Transcript variants 3–7 include all 4 exons. In which, these variants are predicted to produce isoform 3 with a shorter N-terminus compared to isoform 1. Transcript variants 8–10, all produced at the translation start site within exon 4, all translate to protein isoform 4.

Another set of records were analyzed from UniProt, which lists 10 potential MeCP2 isoforms based on P51608 (*MECP2*_HUMAN) [49]. Of these, only the 2 transcripts P51608-1 and P51608-2 are reviewed. P51608-1 and P51608-2 are identical to isoforms 1 (MeCP2E2) and 2 (MeCP2E1), respectively, from NCBI as evident from the alignment (Figure 1). The rest of the isoforms presented in UniProt are computationally predicted. B5MCB4 is translated from the same start codon as MeCP2E1 and hence contains amino acids coded from exon 1 (Figure 1), meaning that exon 2 is spliced out of pre-mRNA. It also contains amino acids translated from exon 3 and part of exon 4 (N-terminus), while the C-terminus is replaced by an alternative fragment that is not matching with any portion of the reviewed MeCP2E1 and E2 sequences. As evident from transcript mapping to the human genome (Supplementary Figure S1), this fragment comes from the translation of exon 4 at a different site. Interestingly, A0A0D9SFX7 has the same C-terminus end fragment as B5MCB4, but it is analogical to MeCP2E2 as it has an amino acid sequence encoded from exon 2. C9JH89 and A0A0D9SEX1 are both missing their reference C-termini starting from the amino acids encoded in exon 3. Their transcripts also have different alternative mRNA 5′ UTR coming from intron 1 of the *MECP2* gene (Supplementary Figure S1). A0A1B0GTV0 is composed of protein fragments matching to N-terminus and an inner fragment of the exon 4 translation product. A0A6Q8PHQ3 and A0A6Q8PF93 have amino acid products from exon 2 only and different alternative C-termini not matching with other protein sequences. Based on the genomic mapping, these fragments are translated from intron 2 of the *MECP2* gene. H7BY72 is missing an arginine from the sequence encoded from exon 2 while having an alternative C-terminus fragment with the same 14 amino acids as A0A6Q8PF93. The UniProt human MeCP2 isoforms and respective Ensembl transcripts are summarized in Table 2.

Among the eight computationally mapped MeCP2 isoforms from UniProt, all but H7BY72, 0A6Q8PHQ3, and A0A6Q8PF93 provide a clear statement of experimental evidence at the protein level (partial/complete Edman sequencing, mass spectroscopy, NMR structure, or X-ray). UniProt labels C9JH89 and A0A0D9SEX1 as coding sequence (CDS) 3′ incomplete, and A0A1B0GTV0 as both CDS 5′ and 3′ incomplete. 

This is also consistent with the alignment, as these proteins are missing portions or complete amino acid sequences corresponding to some of the exons. Based on UniProt records, H7BY72, A0A6Q8PHQ3 and A0A6Q8PF93 are the products of nonsense-mediated decay of isoforms 1 and 2. 

Respective Ensembl transcripts for B5MCB4, C9JH89, A0A1B0GTV0, and A0A0D9SEX1 have TSL of 5 (TSL:5). According to Ensembl Transcript Flags Code, TSL:5 means that there is no single transcript that would support their exon combination model structure. A0A0D9SFX7 and H7BY72 transcripts have support for their exon splicing model from 1 expressed sequence tag (EST) (TSL:3). MeCP2E1 and E2 (P51608-2 and -1) have the strongest TSL of 1, suggesting that all splice junctions of the transcript are supported by at least one non-suspect mRNA.

The functional domains and interaction sites with other proteins of the isoforms were also analyzed and summarized in Table 3 and Table 4. MeCP2 isoform 3 have all but the HMGD1 domain. Isoform 4 on the other hand was found to be missing or having incomplete most of the functional domains (HMGD1 and 2, MBP, aDBD, TRD, NLS1, AT-hook 1, histone H3 methyltransferase interaction sequence (H3-M-T IS). UniProt isoforms were found to be less functionally potential as they are missing most of the essential functional domains and interaction sites required for MeCP2 function. B5MCB4 and A0A0D9SFX7 both have H3-M-T interaction sequence, while missing or having incomplete the rest of the functional features. The rest of the isoforms from UniProt were found to be missing all the functional domains and features.

### 2.2. Histone Post-Translational Modification Profiles at the MECP2 Gene Loci and the Corresponding Cis-Regulatory Elements

**Figure 2:**
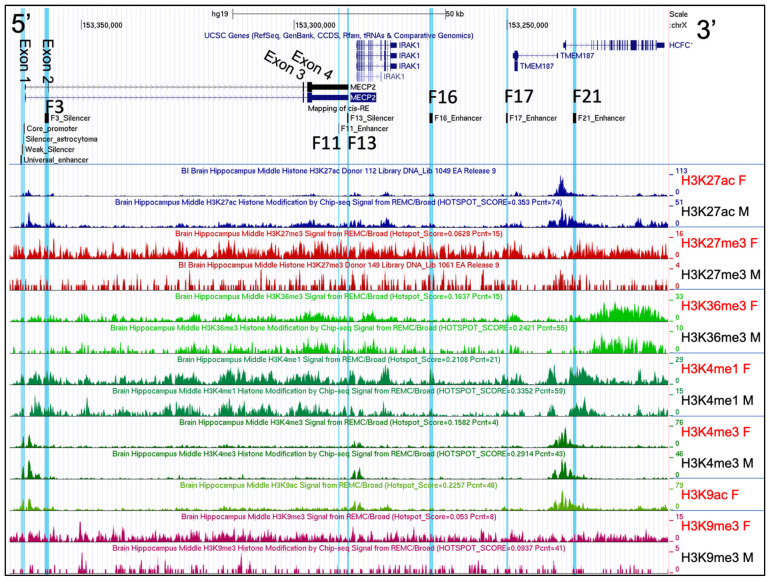
Human Adult Hippocampus histone post-translational modification profiles in males and females. The chrX:153,212,176–153,366,832 genomic region of hg19 human genome assembly is displayed in UCSC genome viewing browser. Direction is 5′–3′ (sense orientation relative to the *MECP2* gene). Tracks displayed from top to bottom: UCSC genes, mapping of previously identified [55] *cis*-regulatory elements (REs) (F3-F21) to genome, 13 histone modification CHIP-seq data tracks. M: male, F: female. *Cis*-REs were highlighted in blue in UCSC. Scale on top represents distance equivalent to 50 kb. *MECP2* exons and F regulatory elements are labelled; F regulatory elements are also highlighted in blue. Histone PTMs data are from GSE17312. The donors selected were: #112 (75 Y, female, disease-free, Rush University Medical Center), and #149 (81 Y, male, disease-free, Rush University Medical Center).

**Figure 3:**
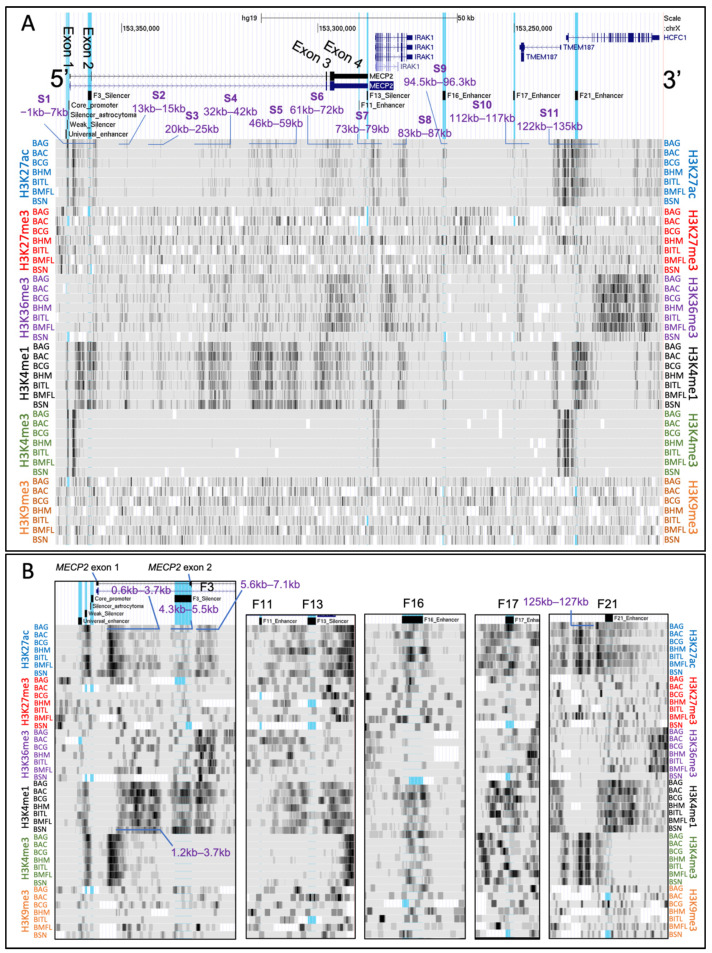
Histone post-translational modification profiles of 7 brain regions. (**A**) The chrX:153,212,176–153,366,832 genomic region of hg19 human genome assembly is displayed in UCSC genome viewing browser. Direction is 5′–3′ (sense orientation relative to the *MECP2* gene). Tracks displayed from top to bottom: UCSC genes, mapping of previously identified [55] *cis*-REs (F3-F21) to genome, histone modification CHIP-seq data tracks. *Cis*-regulatory elements (REs) are highlighted in blue in UCSC. Scale on top represents distance equivalent to 50 kb. *MECP2* exons are labeled on UCSC Genes data track. Genomic segments with consistently high H3K27ac and H3K4me1 were labeled S1–S11. The distances are relative to the first nucleotide of *MECP2* exon 1 (hg19 chrX:153,363,188). Labels consist of 3 letter code for brain region followed by histone PTM type. BAG: brain angular gyrus, BAC: brain anterior caudate, BCG: brain cingulate gyrus, BHM: brain hippocampus middle, BITL: brain inferior temporal lobe, BMFL: brain mid frontal lobe, BSN: brain substantia nigra. Darker areas represent more mapping reads binding to that specific genomic region, hence higher abundance of certain histone PTM. (**B**) Zoomed in portion of the genome focused on the *MECP2* promoter and *cis*-REs. *Cis*-REs are labeled inside and on top of each box. Histone PTMs data are from GSE17312.

Comparative analysis of histone post-translational modifications (PTMs) (H3K27ac, H3K36me3, H3K4me1, and H3K4me3) profiles of the *MECP2* gene and corresponding *cis*-regulatory elements were analyzed from 7 human brain regions: the angular gyrus, anterior caudate, cingulate gyrus, middle hippocampus, inferior temporal lobe, mid frontal lobe, and substantia nigra. These data indicated a high degree of similarity in histone PTMs associated with the *MECP2* gene loci in human male and female tissues (Figure 2 and Figures S2). Limitation of this analysis was exclusion of H3K27ac (substantia nigra), H3K27me3 (cingulate gyrus, mid frontal lobe, angular gyrus), and H3K9ac (all brain regions) in male-female comparisons due to the unavailability of the corresponding datasets. H3K9me3 and H3K27me3 appear to be scattered evenly across the analyzed chromosomal regions and share no distinguishable enrichment peak patterns, when compared between female and male brain tissues. The comparison of multiple histone PTMs between donors of different sexes in 7 different brain regions (Figure 3 and Figure S2) revealed a high degree of similarity between human males and females. We then used data from male tissues to assess the differences in histone PTMs between the 7 brain regions.

We visually assessed the density graphs on hg19 chrX:153,212,176–153,366,832 including the whole *MECP2* gene and all its previously identified *cis*-regulatory elements. We report 7 *MECP2* intragenic segments and 4 segments upstream of the *MECP2* gene, which are highly abundant in H3K27ac and H3K4me1 PTMs (Figure 3). Their estimated positions relative to the *MECP2* transcription start site are (-1)–7 kb (S1), 13–15 kb (S2), 20–25 kb (S3), 32–42 kb (S4), 46–59 kb (S5), 61–72 kb (S6), 73–79 kb (S7), 83–87 kb (S8), 94.5–96.3 kb (S9), 112–117 kb (S10), and 122–135 kb (S11). Out of these: S1-S7 are *MECP2* intragenic; S1 includes *MECP2* proximal and distal promoters, and the F3 silencer. S7 includes *MECP2* F11 enhancer, *MECP2* F13 silencer, *IRAK1* promoter and a 5′ fragment of this gene. S9 includes *MECP2* F16 enhancer; while S10 includes *MECP2* F17 enhancer, and 3′ of *TMEM187* gene. S11 includes promoters and 5′ regions of the *HCFC1* and *TMEM187* genes and complete *MECP2* F21 enhancer. 

On the scale of 50 kb, the trends in H3K27ac and H3K4me1 abundance appear to have a high degree of similarity, with the exception of 125–127 kb region upstream of the *MECP2* TSS, which is enriched in H3K27ac, H3K4me3 and low in H3K4me1 (Figure 3A,B). Interestingly, H3K36me3 was highly abundant at the S1–S6, and S10 genomic segments, but low at the S7, S8, S9 and S11. Instead, H3K36me3 was highly abundant between S6 and S7; S7 and S8; S8 and S9; and from the 3′ end of S11 in the *HCFC1* gene. H3K4Me3 abundance was high at the S1, S7, and S11 regions, which included promoters of the *MECP2*, *IRAK1* and *TMEM187*/*HCFC1* genes, respectively. H3K27me3 and H3K9me3 modification density peaks were evenly scattered throughout the *MECP2* gene and its *cis*-regulatory elements. There was a consistent increase in H3K27me3 histone PTMs upstream of the *MECP2* universal enhancer in 7 brain regions, but peaks did not converge on a certain genomic fragment and were rather scattered. To closely examine the *MECP2 cis*-regulatory elements, we zoomed in to distinguish smaller features (Figure 3B).

The S1 region can be divided into 4 subsegments: proximal and core promoter, 0.6–3.7 kb, 4.3–5.5 kb, including *MECP2* F3 silencer, and 5.6–7.1 kb based on high enrichment with H3K27ac, H3K36me3, and H3K4me1 in 7 brain regions. H3K4me3 abundance is high only at 0.6–3.7 kb subsegment, while the increase in abundance of H3K4me1 starts at 1.2 kb of 0.6–3.7 kb subsegment. H3K9me3 modification density appears to be similar to H3K36me3 with less obvious patterns due to low read mapping coverage. *MECP2* universal enhancer was found to be rich in H3K4me1 in all brain regions analyzed. *MECP2* weak silencer has a high abundance of H3K27ac, H3K4me1, and H3K4me3 in 7 brain regions. *MECP2* universal enhancer and weak silencer are also enriched with H3K9me3, though there is no consistency in modification sites between 7 genomic regions. Silencer of astrocytoma was found to be modified with H3K27ac and H3K4me3 while having a comparatively low abundance of H3K4me1 in 7 brain regions analyzed. The abundance of all histone modifications analyzed was found to be the lowest in *MECP2* core promoter and exon 1 when compared to the nearby genomic regions in all brain regions of interest. 

As discussed previously, *MECP2* F3 silencer was included in one of the S1 subsegments 4.3–5.5 kb upstream of the *MECP2* TSS and was found to be highly enriched with H3K27ac, H3K4me1, and less so with H3K36me3 and H3K9me3 in 7 brain regions analyzed (Figure 3B). F11 enhancer and F13 silencer of *MECP2* show reduction of H3K27ac, H3K36me3, and H3K4me1 even though the nearby genomic regions are highly enriched with these histone PTMs in 7 brain regions. F16 enhancer and F17 silencer of *MECP2* are found to be enriched with H3K27ac, H3K4me1 and H3K4me3, while no distinguishable patterns of increased abundance can be observed for H3K27me3 or H3K9me3 in 7 brain regions. *MECP2* F21 enhancer has increased abundance of H3K27ac, H3K36me3, and H3K4me1. Interestingly, the 125–127 kb subsegment of S11 shows distinctive mutual exclusion of H3K4me1 and H3K4me1, while also being enriched with H3K27ac.

There has also been observed a clear difference in the conservation of histone modification sites between brain regions except for the downstream and upstream proximal to promoter regions of the *MECP2* and other genes.

### 2.3. Chromatin Availability Profiles of the MECP2 Gene and its Cis-Regulatory Elements Characterized by DNase-seq and ATAC-seq in Different Human Tissues and Brain Regions

To characterize chromatin accessibility of the *MECP2* gene and its *cis*-regulatory elements, we aligned DNase-seq datasets from 12 different human tissue samples (adrenal gland, brain, kidney, leg muscle, placenta, spinal cord, stomach, heart, large intestine, left and right lungs, thymus) from both male and female fetuses as well as ovary and testis tissues. As for the histone modification analysis, we first focused on general chromatin accessibility trends within the *MECP2* and other upstream genes which included the *MECP2 cis*-REs (hg19 chrX:153,212,176–153,366,832). 

**Figure 4:**
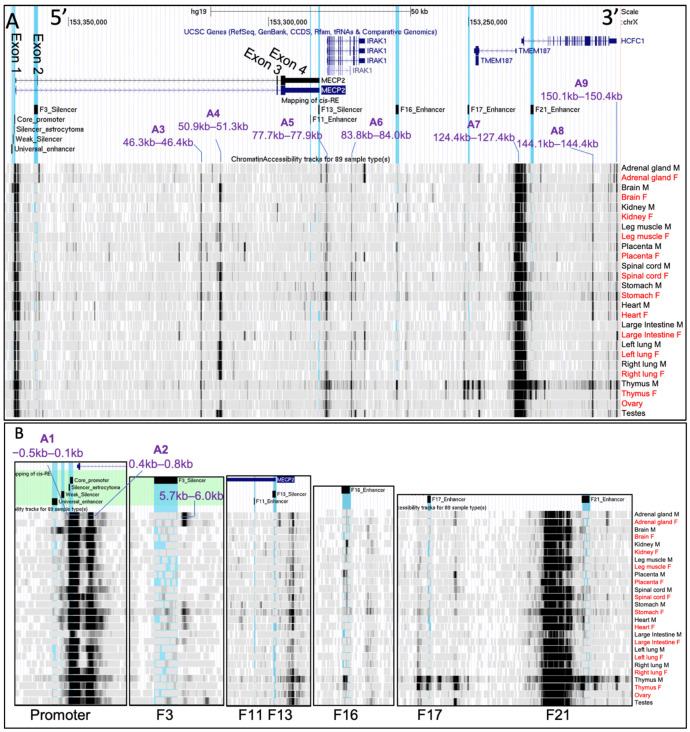
DNase-seq of different fetal tissues: males and females. (**A**) The chrX:153,212,176–153,366,832 genomic region of hg19 human genome assembly is displayed in UCSC genome viewing browser. Direction is 5′–3′ (sense orientation relative to the *MECP2*) gene. Tracks displayed from top to bottom: UCSC genes, mapping of previously identified [55] *cis*-REs (F3-F21) to genome, DNase-seq data tracks. *Cis*-REs were highlighted in blue in UCSC. Scale on top represents distance equivalent to 50 kb. *MECP2* exons are labeled on UCSC Genes data track. Genomic segments with consistently high chromatin accessibility (darker regions) are labeled A1–A9. The distances are relative to the first nucleotide of the *MECP2* exon 1 (hg19 chrX:153,363,188). Labels are copied and added to the left for easier reference. Labels consist of a tissue type followed by M (male) or F (female). Darker areas represent more mapping reads binding to that specific genomic region, hence higher chromatin accessibility. (**B**) Zoomed in portion of the genomes focused on the *MECP2* promoter and *cis*-REs. *Cis*-REs are labeled inside and on the top of each box. Labels are the same as in pane A. DNase-seq data included data tracks from various donors of 85 to 127 days old during the fetal stage of development from data series GSE18927.

On the scale of 50 kb, we report 9 genomic regions (Figure 4A) which have consistently higher chromatin accessibility compared to the nearby regions in most of the studied tissues in both sexes, which we will refer to as A1–A9, of which A1–A4 are *MECP2* intragenic: A1 (−0.5–0.1 kb) includes *MECP2* silencer of astrocytoma, core promoter, and exon 1 (Figure 4B); A2 (0.4–0.8 kb) is a fragment of *MECP2* intron 1 (Figure 4B); A3 (46.3–46.4 kb) and A4 (50.9–51.3 kb) are fragments of *MECP2* intron 2; A5 (77.7–77.9 kb) includes *IRAK1* promoter; A6 (83.8–84.0 kb) is *IRAK1* intragenic fragment; A7 (124.4–127.4 kb) includes promoters of *TMEM187* and *HCFC1* as well as their exons 1 and introns 1; A8 (144.1–144.4 kb) and A9 (150.1–150.4 kb) are intragenic *HCFC1* fragments in intron 18 and exon 26. Outside of A1–A11, conservation of accessible regions is highly reduced between different tissues, while there is a low degree of similarity between sexes within each tissue. Both female and male thymus tissue appear to have the highest average accessibility of chromatin in the analyzed regions.

Then, we assessed chromatin accessibility at each of the *MECP2 cis*-regulatory elements (Figure 4B). *MECP2* universal enhancer has an increased chromatin accessibility only in male brain tissue, with only some accessibility increase in females. Other tissues exhibit variable accessibility at universal enhancer and no apparent conclusion about differences between sexes or different tissues can be made. *MECP2* silencers of astrocytoma, core promoter, and exon 1 appear to be highly accessible in all tissues of both sexes. *MECP2* F3 silencer chromatin shows some degree of accessibility in the spinal cord, stomach, heart in both sexes and testes. 5.7–6.0 kb fragment (upstream of *MECP2* exon 2 and F3 silencer) shows a high degree of chromatin accessibility in both female and male adrenal glands, stomach, and heart with lower accessibility in thymus, ovary, and testes. *MECP2* F11 enhancer and F13 silencer show no signs of chromatin accessibility and seem to have low read mapping densities. F16 enhancer of *MECP2* was found to be highly accessible in the kidney, placenta, and thymus of both sexes, with lower accessibility in the adrenal gland, spinal cord, and stomach. F17 and F21 enhancers of *MECP2* are highly accessible in the thymus of both sexes. F21 is also distinguishably more accessible in the male brain, and both male and female spinal cords, stomach, heart, and large intestine.

**Figure 5:**
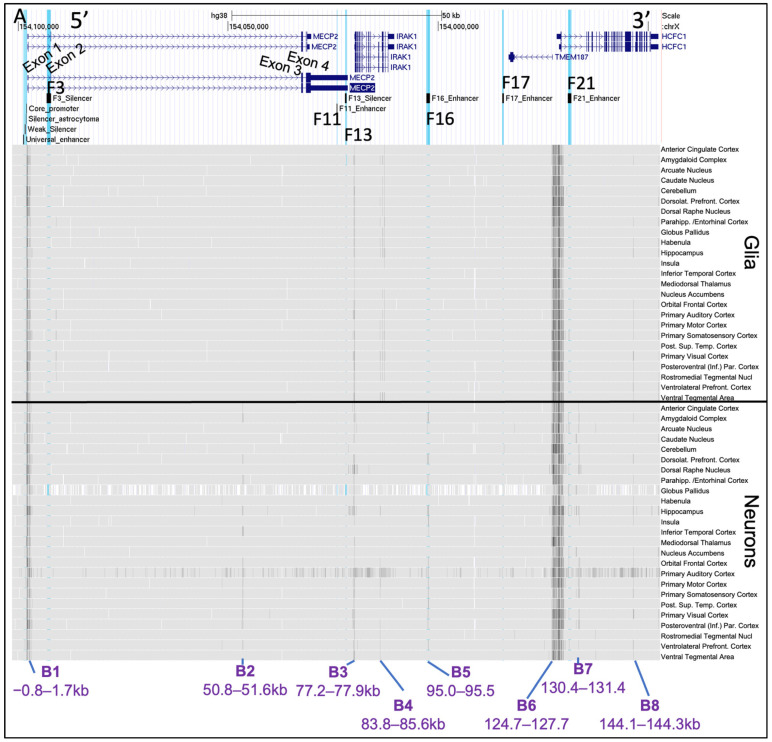

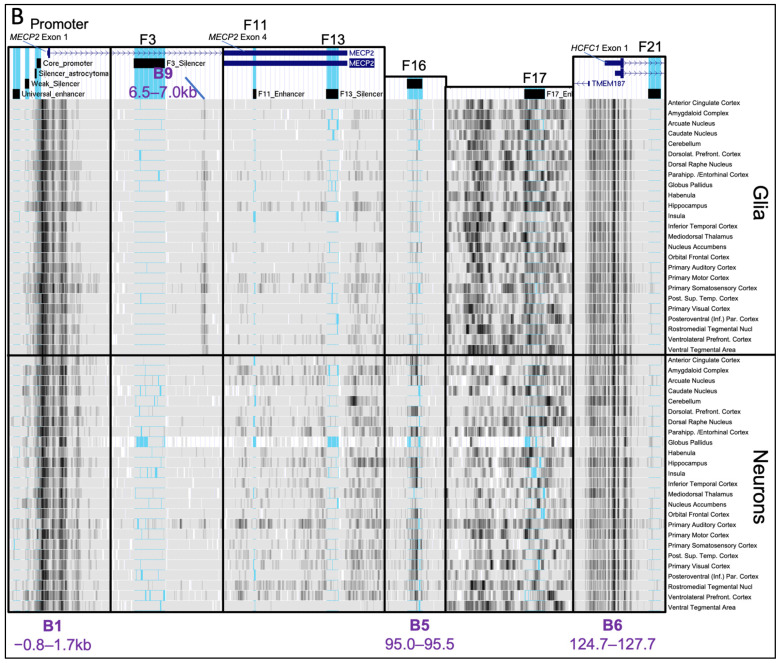
ATAC-seq of 25 brain regions: glia and neurons. (**A**) The chrX:153,947,013–154,101,440 genomic region of hg38 human genome assembly is displayed in UCSC genome viewing browser. Direction is 5′–3′ (sense orientation relative to the *MECP2* gene). Tracks displayed from top to bottom: ENCODE genes, mapping of previously identified [55] *cis*-REs (F3-F21) to genome, ATAC-seq data tracks. *Cis*-REs are highlighted in blue in UCSC. Scale on top represents distance equivalent to 50 kb. *MECP2* exons are labeled on ENCODE Genes data track. Genomic segments with higher chromatin accessibility (darker regions) were labeled B1–B9. The distances are relative to the first nucleotide of *MECP2* exon 1 (hg38 chrX:154,097,717). Labels are replaced for better readability. The labels define glial and neural cells from the 25-brain region analyzed Darker areas represent more mapping reads binding to that specific genomic region, hence higher chromatin accessibility. (**B**) Zoomed in portion of the genomes focused on the *MECP2* promoter and *cis*-REs. *Cis*-REs are labeled inside and at the bottom of each box. The labels are the same as in panel (**A**). The ATAC-seq data tracks were uploaded from GSE211822.

Next, we assessed chromatin accessibility at the *MECP2* gene promoter, corresponding *cis*-REs, and nearby genomic regions in glia and neurons from 25 brain cortical and subcortical areas from GSE211822 ATAC-seq datasets. High accessibility regions were defined as B1–B9 (Figure 5) and further investigation was performed at a lower scale on each of the previously discovered *cis*-REs and the *MECP2* promoter. 

The results showed that B1 (−0.8–1.7 kb), B3 (77.2–77.9 kb) and B6 (124.7–127.7 kb) are consistently accessible in all brain regions in both glial and neuronal cells (Figure 5A). B1 was found to partially overlap with the *MECP2* promoter elements with highest accessibility levels observed on −0.2–0.1 kb between the core promoter and TSS, and within 5′ proximal end of intron 1 of the *MECP2* gene (0.2–0.3 kb; 0.4–0.7 kb) in all brain regions of glial cells and neurons (Figure 5B). Neuronal cells had more inconsistent levels of accessibility between the brain regions within B1. Differences in accessibility between the brain regions in both neurons and glia were found at the *MECP2* core promoter, silencer of astrocytoma, weak silencer, and universal enhancer. These regions exhibited highly inconsistent chromatin availability between the brain regions and cell types. B3 and B6 might overlap with the *IRAK1* and *HCFC1/TMEM187* promoters, respectively. Evidently, the highest chromatin availability is observed at the *HCFC1/TMEM187* promoters, followed by *MECP2* and then *IRAK1* promoters. B6 exhibits a high level of chromatin accessibility conservation between the brain regions and cell types with a pattern of alternating accessibility throughout the fragments (Figure 5B). B9 (6.5–7.0 kb) was found in the *MECP2* intron 1 downstream of F3 silencer upon closer examination of the *cis*-REs (Figure 5B). B9 exhibits consistently higher chromatin availability in glia from most of the brain regions with only some accessibility in neurons.

With variation between the brain regions, B2 (50.8–51.6 kb/a portion of the *MECP2* intron 2), B5 (95.0–95.5/F16 enhancer), and B7 (130.4–131.4/a portion of the *HCFC1* intron 1) have a higher chromatin availability in neurons and in most brain regions (Figure 5A). B5 overlaps with F16 enhancer (Figure 5B). B4 (83.8–85.6 kb/a portion of the *IRAK1* intron) and B8 (144.1–144.3 kb/a portion of *HCFC1* intron) had inconsistent chromatin availability between the analyzed brain regions and cell types (Figure 5A).

F17 had no distinctive accessibility patterns. F3, F11 and F13 had reduced accessibility in all brain regions in both cell types. F21 exhibited a variable accessibility predominantly higher in neurons than in glia inconsistently between different brain regions.

### 2.4. DNA Methylation of Mid Frontal Gyrus at Different Stages of Human Development

**Figure 6:**
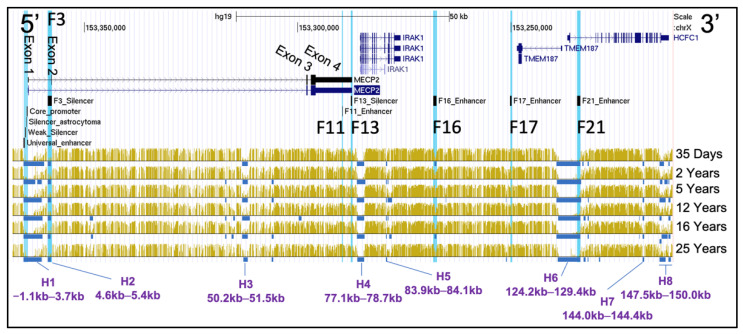
BS-seq of mid-frontal gyrus at different ages. The chrX:153,212,176–153,366,832 genomic region of hg19 human genome assembly is displayed in UCSC genome viewing browser. Direction is 5′–3′ (sense orientation relative to the *MECP2* gene). Tracks displayed from top to bottom: UCSC genes, mapping of previously identified [55] *cis*-REs (F3-F21) to genome, BS-seq data tracks: combination of DNA-methyl abundance (yellow) and DNA hypomethylated regions (blue). *Cis*-REs are highlighted blue in UCSC. Scale on top represents distance equivalent to 50 kb. The *MECP2* exons are labeled on UCSC Genes data track. Hypomethylated genomic segments which were consistent between the ages (darker regions) were labeled H1–8. The distances are relative to the first nucleotide of the *MECP2* exon 1 (hg19 chrX:153,363,188). Labels include donor age. Yellow peaks represent more mapping reads binding to that specific genomic region, hence higher abundance of methyl-DNA. The methylation data of frontal gyrus of different donors’ ages are from GEO: GSE17312.

Next, we assessed DNA methylation of the genomic region hg19 chrX:153,212,176–153,366,832, containing the *MECP2* and other upstream genes with the *MECP2 cis*-REs. BS-seq data [45] of healthy male human mid frontal gyrus were compared between 6 donors of different ages: 35 days, 2 years, 5 years, 12 years, 16 years, and 25 years of age (Figure 6). We report 8 hypomethylated genomic segments analyzed on the X chromosome, which will be referred to as “H1–H8”. These segments are consistently hypomethylated at all 6 ages. H1(−1.1–3.7 kb) includes all the *MECP2* promoter regulatory elements (universal enhancer, weak silencer, the silencer of astrocytoma, core promoter, and a portion of *MECP2* intron 1 (0–3.7 kb). H2 (4.6–5.4 kb) aligns with the *MECP2* F3 silencer. H3 (50.2–51.5 kb) is within the *MECP2* intron 2. H4 (77.1–78.7kb) includes *IRAK1* promoter and 5′ intra genic fragment (exons 1–4, introns 1–3). H5 (83.9–84.1kb) is the *IRAK1* intron 11 fragment. H6 (124.2–129.4 kb) includes the *TMEM187* and *HCFC1* promoters and 5′ intragenic regions (exons 1 and introns 1 of both genes), as well as the *MECP2* F21 enhancer. H7 (144.0–144.4 kb) was found to be a part of intron 18 of the *HCFC1* gene. H8 (147.5–150.0 kb) segment is composed of multiple hypomethylation subsegments variable between the analyzed ages, and it included exons 25–26 and intron 25 of the *HCFC1* gene. F11, F16, F17 enhancer or F13 silencer were not found to be hypomethylated. Within the *MECP2* gene, there are some differences in the locations of the hypomethylated regions in intron 2 between the 6 ages, however, it is not clear if this is due to difference in methylation density or insufficient genome mapping reads coverage as the overall methylation profiles between the different ages show a high degree of similarity. 

## 3. Discussion

### 3.1. Potential MeCP2 Protein Isoforms

Several previous studies have provided evidence for a difference in relevance of known MeCP2E1 and MeCP2 E2 isoforms in neurodevelopment and associated disorders [53,54,56]. Analysis of potential MeCP2 isoforms is essential for the understanding of all MeCP2 functions and its complete role in the development of different organs. 

NCBI lists 10 reviewed transcripts that are predicted to encode 4 protein isoforms, 2 of which are MeCP2E1 and MeCP2E2. The other 2 isoforms, isoforms 3 and 4, have shorter N-termini than E1 or E2 (Figure 1). UniProt lists 10 proteins isoforms, 2 of which are MeCP2E1 (P51608-2) and MeCP2E2 (P51608-1). 

Alignment of both NCBI and UniProt MeCP2 protein isoforms provides insights into potential functions of the isoforms if they exist in vivo. Functional domains are defined as minimal protein regions required for certain protein functions; therefore, we can assume that if only a part of a defined functional domain is present, the respective protein function is expected to be reduced or eliminated. As expected, NCBI isoforms 1 and 2 and UniProt isoforms P51608-1 and -2 have all of the functional domains as these are MeCP2E2 and MeCP2E1, respectively. The rest of potential MeCP2 isoforms were found to be missing complete or portions of their functional domains or interaction sites with other proteins (Table 3 and Table 4) potentially suggesting their limited functions. NCBI MeCP2 isoform 3 was missing HMGD1 and had incomplete methyl-CpG-binding domain (MBD) and H3-M-T interaction sequence, suggesting that this isoform could still be transported to the nucleus, and may have the capability of binding to DNA non-specifically, remodel chromatin, repress transcription, and interact with histone H3 methyltransferase (H3-M-T), NCoR/Smrt co-repressor, TBL1XR1, and group 2 WW motif-containing protein. NCBI MeCP2 isoform 4 is missing or has incomplete functional domains except for NLS2 and AT-hook 2, and it also has all of the protein interacting sites, besides H3-M-T. Isoform 4 is predicted to mostly function as a mediator for other transcription repressing proteins (NCoR/Smrt co-repressor, TBL1XR1, and group 2 WW motif-containing protein) by binding to DNA non-specifically with its AT-hook 2. Since C-terminal domain (CTD) is present, isoform 4 is also potentially expected to participate in the assembly of chromatin structures. All of the predicted isoforms from UniProt were found to be missing most of their functional features including NLS1 and 2, suggesting that even if they existed in vivo, they would have little to no effect on transcription as they would not be transported into the nucleus after translation.

### 3.2. In Silico Analysis of the Human MECP2 Gene

MeCP2 isoforms are known to exhibit differential expression between brain regions [36] and between different brain cell types [34] in mice. Despite the importance of MeCP2 expression regulation during brain development, the mechanisms through which MeCP2 expression is regulated are not well defined. In this part of the study, we produced *MECP2* gene epigenetic maps to characterize *MECP2* gene promoter and *cis*-REs in different brain regions and tissues of human donors of various ages and sexes. We focused on previously described *MECP2 cis*-REs [55] and applied in silico methods for data mining and visualization of datasets using the UCSC genome viewing browser. 

**Figure 7:**
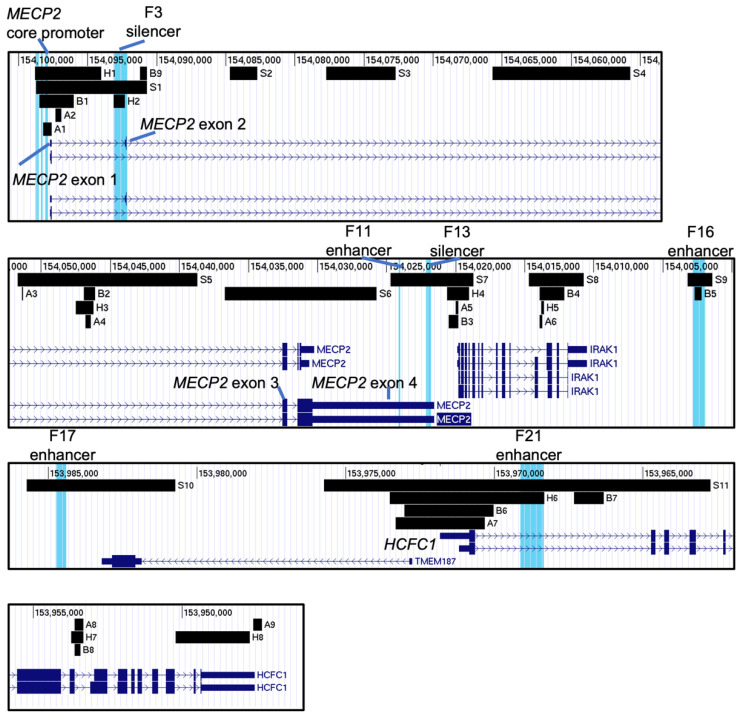
Summary of the identified genomic segments potentially involved in regulation of the *MECP2* gene. The identified genomic segments of interest were added to the hg38 human genome in UCSC genome viewing browser. Previously published F elements were highlighted blue and labelled on top of each pane. “S” fragments: histone PTMs in various brain regions (Figure 3), “A” fragments: highly accessible chromatin in different fetal tissues based on DNA-seq (Figure 4), “B” fragments: highly accessible chromatin in different brain regions of adults based on ATAC-seq (Figure 5), “H” fragments: hypomethylated genomic regions of mid-frontal gyrus in individuals 35d to 25 years old (Figure 6). Direction is 5′–3′ (sense direction relative to the *MECP2* gene).

All the genomic fragments identified in this study were overlayed in UCSC to assess the relation between different epigenetic modifications and chromatin accessibility (Figure 7). It can be observed that the distinct epigenetically significant fragments identified in this study tend to co-localize. This was expected, considering that the nucleosome free regions would contain binding sites for various transcription factors. It was also anticipated that the gene promoters would exhibit more open chromatin structure and epigenetic modifications associated with euchromatin, suggesting their essential role in transcription initiation. Previously identified F-elements have been further characterized. However, we also observed co-localization of “A”, “B” and “H” fragments at the A4 (*MECP2* intron 2), A6 (*IRAK1* intron) and A8 (*HCFC1* intron) suggesting that they are hypomethylated and are accessible throughout different tissues.

H3K27ac in tandem with H3K4me1 defines active enhancers [57]. An increase in these histone PTMs is observed in F16, F17, F21 enhancers, and F3 silencer in various human brain regions. Even despite the confirmed function of F3 as a silencer of *MECP2,* its histone modification profile fits enhancer criteria. At the same time, F3 and F21 were found hypomethylated in mid-frontal human gyrus of different age, while F16 was found to be accessible (fragment B5) throughout the neural and glial cells of various parts of adult brain. F13 silencer was found to have a lower abundance of H3K27ac and H3K4me1 when compared to the nearby genomic regions. H3K4me3 is known to be highly abundant at the transcription start sites [58], which is consistent with what we observe in Figure 3B. 

H3K4me3 is also highly abundant upstream of the *MECP2* TSS in intron 1 (~0.6–1.2 kb), which in combination with H3K27ac and H3K36me3 [59] may facilitate alternative splicing of the *MECP2* isoforms. This segment was also found to be hypomethylated in mid-frontal human gyrus of different age (H1) and accessible in different tissues (A1, A2) and throughout the brain (B1). H3K36me3 which associates with euchromatin differs in regions of high abundance from H3K4me1 and H3K27ac mostly outside of the *MECP2* gene, potentially indicating its possible co-acting role with other modifications within the *MECP2* gene. H3K27me3 is associated with a long-term silencing of genes [60] via the formation of the facultative heterochromatin. H3K9me3 is known to be associated with constitutive heterochromatin [60]. We report even distribution for both of the transcription repressive modifications (H3K27me3, H3K9me3) throughout the *MECP2* gene and its *cis*-REs suggesting on the activity level of the *MECP2* gene in these analyzed 7 brain regions. H3K4me1 and H3K4me3 are found to be almost mutually exclusive, suggesting their positional restriction to outside and within the promoter, respectively. 

Some previously identified enhancers and silencers exhibited similar chromatin accessibility profiles. Even though this information does not provide any insights regarding *MECP2* expression in different tissue types and cellular subtypes, these findings may suggest the presence of antagonistic effect of various active *cis*-REs on the *MECP2* expression. 

## 4. Materials and Methods

### 4.1. Analysis of MECP2 Transcripts and Protein Isoforms from NCBI, UniProt, ENSEMBL

*MECP2* human gene was identified in the NCBI database (Gene ID: 4204). GenBank records for all 10 transcripts relevant to the 4204 gene were extracted as a .gb file. From the file, XM_ sequences were identified and excluded from this study as being non-reviewed. “Comment” sections of each record were analyzed to identify MeCP2E1 (also known as MeCP2α or MeCP2B) and MeCP2E2 (also known as MeCP2β or MeCP2A) coding transcripts. Information about the difference between *MECP2E1/E2* and other transcripts as well as encoded protein isoforms was also extracted from the “Comment” sections. “Evidence-Data” codes were interpreted using Evidenceontology.org [61]. NCBI respective protein sequences encoded by 10 transcripts were extracted as a .gp file. UniProt record for human MeCP2 protein (P51608) has also been analyzed. Eight potential protein isoform record sequences and 2 reviewed sequences were extracted from the record. Additionally, from each potential isoform record, Ensembl cross-references were extracted from the “Genome annotation databases” section. Then, Ensembl transcript support level was used to assess the quality of the sequences. Then, selected sequences were aligned using ClustalOmega [62] multiple sequence alignment web tool. Exon nucleotide sequences were retrieved from NG_007107.3 human *MECP2* record and translated using Expasy “Translate” tool [63]. Then, the translated exons sequences from the appropriate reading frames were highlighted in the alignment. The following functional groups were also labelled on the alignment: methyl-CpG-binding domain (MBD) sequence [64,65], transcriptional-repression domain (TRD) [55], high mobility group-like domains 1 and 2 (HMGD1 and 2) [65], nuclear localization signals 1 and 2 (NLS1 and 2) [65], alternative DNA binding domain (aDBD) [65], group 2 WW motif-containing protein interaction region (WW-2) [65], histone H3 methyltransferase interaction region [65], two AT hooks (from UniProt P51608 record), TBL1XR1 interaction region (from UniProt P51608 record), NCoR/SMRT interaction domain (NID) [22], C-terminal domains α and β (CTDα and CTDβ) [65] (Supplementary Table S1). To find the origin of the reference non-matching sequences, cDNA of Ensembl transcript sequences, from which UniProt isoforms were derived, was used as a query for UCSC BLAT [66] along with the reference exon sequences for the human *MECP2* gene extracted from NCBI. The output of BLAT was visualized in the UCSC genome viewing browser in the form of mapping of query sequences to the hg38 version of the human genome (Supplementary Figure S1). All information collected from the databases and alignment analysis were summarized in Table 1, Table 2, Table 3 and Table 4. The data were last verified on 15 October 2022.

### 4.2. Locating the MECP2 Gene Regulatory Elements in UCSC

Previously, in 4 human cell lines (HeLa, SK-N-SH, HT1080, and CRL1718) 2 silencer and 4 enhancer elements were identified and functionally confirmed around *MECP2*, as well as at the *MECP2* core and proximal promoter [55]. The human *MECP2* gene was identified in the NCBI database (Gene ID: 4204). Relative to TSS position numbers of the promoter elements were subtracted from the first nucleotide number of exon 1 to find the sequences. F3, F11, F13, F16, F17, F21 regulatory elements sequences were extracted from NCBI Nucleotide Database based on their “gi:” accession number and positions of the regions. All the sequences were formatted in fasta and used as input to BLAT [66] to be aligned with Human hg19 genome assembly. This assembly was chosen as the one having the largest number of datasets available. Then, the alignment was visualized in UCSC as a custom track.

### 4.3. Selection and Visualization of Datasets in UCSC

UCSC genome viewing browser [50] was used to visualize whole-genome sequencing data files deposited from NCBI Gene Expression Omnibus (GEO) [67] and ENCODE [68,69] databases. 

Roadmap Epigenomics Visualization Hub, collection of Roadmap Epigenomics Project data files (also available in GEO [67]: Series GSE17312), was used to search for available whole-genome sequencing datasets pre-uploaded to UCSC file collection. A part of the information about data tracks selected is labelled on top of each track in UCSC. For histone PTMs analysis, the donors selected were: #112 (75 Y, female, disease-free, Rush University Medical Center), and #149 (81 Y, male, disease-free, Rush University Medical Center). All available data for histone PTMs were included in the analysis. Multiple brain region histone PTMs analysis alignment was created using all data from male data sets used for sex comparison figures. 

The analysis of DNase-seq data included data tracks from various donors of 85 to 127 days old during the fetal stage of development from The NIH Roadmap Epigenomics Mapping Consortium data series (GSE18927). The representative replicates were selected to be close in age between male and female tissue samples. Tissue selection was based on the presence of both male and female tissue datasets available in UCSC.

ATAC-seq data was added to UCSC Human hg38 genome from GSE211822 submitted by Fullard J.F., Dong P., Bendl J., and Roussos P. as custom data tracks. Both glial and neuronal cells from 25 brain cortical and subcortical areas were included from 6 neurotypical controls as per the data series description section. 

The analysis of methylation data of frontal gyrus of different donors’ ages is from GEO: GSE17312 [67,70], which had also been pre-uploaded to UCSC. Processed bisulphate sequencing data was displayed in 6 data tracks as the mean value DNA methylation on the scale from 0 to 1 (limited by black lines). The other 6 data tracks highlight hypomethylated regions (HMRs) in blue. All tissue samples are from healthy males of respective ages as labelled.

All data tracks were configured as follows: type of graph = bar, data view scaling = auto-scale to data view. The genome sequence was reversed so that *MECP2* exon 1 is on the left. Other settings were kept as default. Relative distances to a TSS were determined by subtracting the first nucleotide position of *MECP2* exon 1 (including 5′ UTR) from the first and last nucleotide positions of the genomic segment of interest. Upstream and downstream sequences relative to TSS were assigned positive and negative values, respectively. Mapped *cis*-REs were highlighted in UCSC to find the corresponding fragments on the data tracks. 

The links to the UCSC sessions with the described above data tracks are included as Supplementary Table S2.

## 5. Conclusions

Based on the support level, sequence alignments and identification of which functional domains are present in each isoform, we report NCBI MeCP2 isoforms 3 and 4 have the potential for functional existence in vivo, unless they are degraded at the transcript level. We hypothesize that if they existed in vivo, they would have required functional domains to bind to DNA non-specifically, reconfigure chromatin, repress transcription and interact with other proteins. As a future direction, the presence and the expression levels of these transcripts need to be confirmed in vivo and in vitro, along with their protein levels and their stability.

We identified genomic fragments with distinct patterns of histone PTMs, hypomethylation, and DNA accessibility in various human tissues and cell types. In addition to their co-localization in promoter regions, we also described other intronic potential areas of interest. Overall, despite high degree of similarity, the differences in these epigenetic profiles might result in previously reported differential *MECP2* expression in different tissues and brain regions. 

## Figures and Tables

**Table 1:** Summary of NCBI *MECP2* mRNA variants and their respective protein isoforms. (Updated 15 October 2022).

Accession Number	Transcript Variant	Protein Isoform Encoded
NM_004992.4	1	Translation initiates in exon 2, producing protein isoform 1 with a distinct N-terminus
NM_001110792.2	2	Translation initiates in exon 1, producing protein isoform 2 with a distinct N-terminus
NM_001316337.2	3	Product: Protein isoform 3; it has a shorter N-terminus compared to isoform 1
NM_001369391.2	4	
NM_001369392.2	5	
NM_001369393.2	6	
NM_001369394.2	7	
NM_001386137.1	8	Product: Protein isoform 4
NM_001386138.1	9	
NM_001386139.1	10	

**Table 2:** Summary of UniProt MeCP2 protein isoform variants and their respective transcripts from Ensembl (Updated 15 October 2022).

UniProt Data			Ensembl Data			
UniProt Identifier	Status	Evidence	Ensembl Transcript ID	Biotype	Transcript Support Level (TSL)	Incomplete
P51608-1	Reviewed	Evidence at protein level	ENST00000303391.11	Protein coding	TSL:1	-
P51608-2	Reviewed	Evidence at protein level	ENST00000453960.7	Protein coding	TSL:1	-
B5MCB4	Unreviewed	Evidence at protein level	ENST00000407218.5	Protein coding	TSL:5	-
A0A0D9SFX7	Unreviewed	Evidence at protein level	ENST00000628176.2	Protein coding	TSL:3	-
C9JH89	Unreviewed	Evidence at protein level	ENST00000415944.3	Protein coding	TSL:5	CDS 3’ incomplete
A0A1B0GTV0	Unreviewed	Evidence at protein level	ENST00000637917.1	Protein coding	TSL:5	CDS 5’ and 3’ incomplete
A0A0D9SEX1	Unreviewed	Evidence at protein level	ENST00000630151.2	Protein coding	TSL:5	CDS 3’ incomplete
H7BY72	Unreviewed	Protein predicted	ENST00000369957.5	Nonsense mediated decay	TSL:3	-
A0A6Q8PHQ3	Unreviewed	Protein predicted	ENST00000674996.1	Nonsense mediated decay	-	-
A0A6Q8PF93	Unreviewed	Protein predicted	ENST00000675526.1	Nonsense mediated decay	-	-

**Table 3:** Summary of the functional domains present in predicted protein isoforms from NCBI (Updated 15 October 2022).

	NCBI			
	Isoform 1	Isoform 2	Isoform 3	Isoform 4
Exon 1	-	✓	-	-
Exon 2	✓	-	-	-
Exon 3	✓	✓	N-term. incompl. Starts at alternative M in exon 3	-
Exon 4	✓	✓	✓	N-term. incompl. Starts at alternative M in exon 4
HMGD1	✓	Possible different N-terminus	-	-
HMGD2	✓	✓	✓	-
MBD	✓	✓	N-term. incompl.	-
aDBD	✓	✓	✓	N-term. incompl.
TRD	✓	✓	✓	N-term. incompl.
NLS1	✓	✓	✓	-
NLS2	✓	✓	✓	✓
AT hook 1	✓	✓	✓	-
AT hook 2	✓	✓	✓	✓
H3-M-T IS	✓	✓	N-term. incompl.	-
NID	✓	✓	✓	✓
TBL1XR1 IS	✓	✓	✓	✓
WW-2 IS	✓	✓	✓	✓
CTD⍺	✓	✓	✓	✓
CTDβ	✓	✓	✓	✓

**Table 4:** NLS1-2, AT-hook 1-2, and CTDβ sequences were not found in any of the potential isoforms from UniProt (Updated 15 October 2022).

	UniProt							
	B5MCB4	A0A0D 9SFX7	C9JH89	A0A1B0 GTV0	A0A0D 9SEX1	H7BY72	A0A6Q 8PHQ3	A0A6Q 8PF93
Exon 1	✓	-	-	-	-	-	-	-
Exon 2	-	✓	✓	-	✓	C-term. incompl.	✓	✓
Exon 3	✓	✓	C-term. incompl.	-	C-term. incompl.	-	-	-
Exon 4	C-term. incompl.	C-term. incompl.	-	C-term. incompl, missing fragment inside	-	-	-	-
HMGD1	Possible different N-terminus	✓	C-term. incompl.	-	C-term. incompl.	C-term. incompl.	C-term. incompl.	C-term. incompl.
HMGD2	-	C-term. incompl.	-	-	-	-	-	-
MBD	C-term. incompl.	C-term. incompl.	-	N-term. and C-term. Incompl.	-	-	-	-
aDBD	-	-	-	N-term. incompl.	-	-	-	-
TRD	-	-	-	N-term. incompl.	-	-	-	-
H3-M-T IS	✓	✓	-	-	-	-	-	-
NID	-	-	-	N-term. incompl.	-	-	-	-
TBL1XR1 IS	-	-	-	N-term. incompl.	-	-	-	-
WW-2 IS	-	-	-	-	-	-	-	-
CTDα	-	-	-	C-term. incompl.	-	-	-	-
CTDβ	-	-	-	-	-	-	-	-

## Data Availability

Not applicable. All information for this manuscript has been extracted from publicly available datasets.

## Supplementary Materials

The following supporting information can be downloaded at: https://www.mdpi.com/article/10.3390/ijms232415643/s1.

## Abbreviations

5-hmC: 5-hydroxy-methyl cytosine; 5-mC: 5-methyl cytosine; aDBD: alternative DNA binding domain; ATAC-seq: assay for transposase-accessible chromatin with sequencing; BAC: brain anterior caudate; BAG: brain angular gyrus: BCG: brain cingulate gyrus; BHM: brain hippocampus middle; BITL: brain inferior temporal lobe; BMFL: brain mid frontal lobe; BRE: B recognition element; BSN: brain substantia nigra; BS-seq: bisulfite sequencing; CDS: coding sequence; *cis*-REs: *cis*-regulatory elements; CTDα: C-terminal domain α; CTDβ: C-terminal domain β; DNAse-seq: DNase I hypersensitive sites sequencing; DPE: downstream promoter element; EMBL-EBI: European Bioinformatics Institute; EST: expressed sequence tag; GEO: Gene Expression Omnibus; F: female(s); H3-M-T: histone H3 methyltransferase; H3-M-T IS: histone H3 methyltransferase interaction sequence; HDAC: histone deacetylases; HMGD1:high mobility group protein-like domain 1; HMGD2: high mobility group protein-like domain 2; HMR: hypomethylated region; Inr: Initiator element; INSDC: International Nucleotide Sequence Database Collaboration; kb: kilobase; M: male (s); MBD: methyl-CpG-binding domain; MeCP2: Methyl CpG binding protein 2; NCBI: National Center for Biotechnology Information; NID: NCoR/SMRT interaction domain; NLS1: nuclear localization signals 1; NLS2: nuclear localization signals 2; NM_: mRNA; PIR: Protein Information Institute; PTM: post-translational modifications; RefSeq: Reference Sequence; RTT: Rett Syndrome; SIB: Swiss Institute of Bioinformatics; TBL1XR1 IS: TBL1XR1 interaction sequence; TRD: transcriptional repression domain; TrEMBL: Translated EMBL Nucleotide Sequence Data Library; TSS: transcription start site; UniParc: UniProt Archive; UniProt: Universal Protein Resource; UniProtKB: UniProt Knowledgebase; UniRef: UniProt Reference Clusters; WW-2 IS: Group 2 WW motif-containing protein interaction sequence.
