# Supplementary material for: The Chromatin Structure at the MECP2 Gene and In Silico Prediction of Potential Coding and Non-Coding MECP2 Splice Variants
Source: Int J Mol Sci. 2022 Dec 9;23(24):15643. doi: 10.3390/ijms232415643 (PMC9779294; doi:10.3390/ijms232415643)
Supplement: Supplementary file 1 [file ijms-23-15643-s001.zip › ijms-2038051-supplementary.pdf]

Supplementary Figure S1. Mapping of *MECP2* Ensembl transcripts cDNA to the *MECP2* gene.

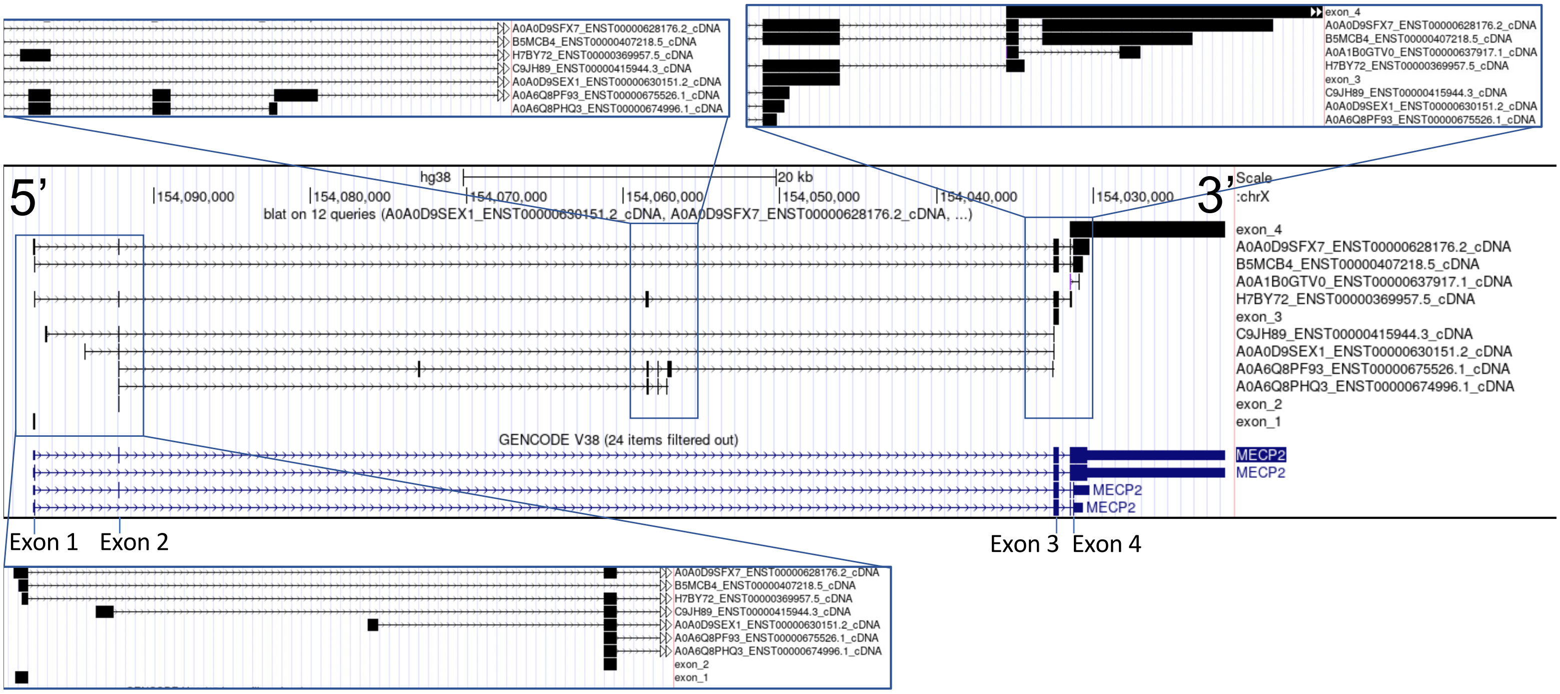


The cDNA sequences from the Ensembl *MECP2* transcripts were mapped to hg38 human genome using BLAT in UCSC. Panes above and below the alignment are zoomed in regions indicated with blue rectangles. Direction of the genomic sequence is 5′ – 3′; sense orientation relative to the *MECP2* gene. Tracks which are displayed from the top to the bottom are: mapping sequences, GENCODE reference sequence. *MECP2* exons are labeled below the central pane. Mapped sequences' IDs are labeled on the right. Black vertical dashes and rectangles represent mapped sequences.

Supplementary Figure S2. (A) Adult Cingulate Gyrus, (B) Mid Frontal lobe, (C) Adult Anterior Caudate, (D) Adult angular gyrus, (E) Adult Substantia Nigra, and (F) Adult Inferior Temporal Lobe Histone PTMs of Males and Females.

(A) Adult Cingulate Gyrus

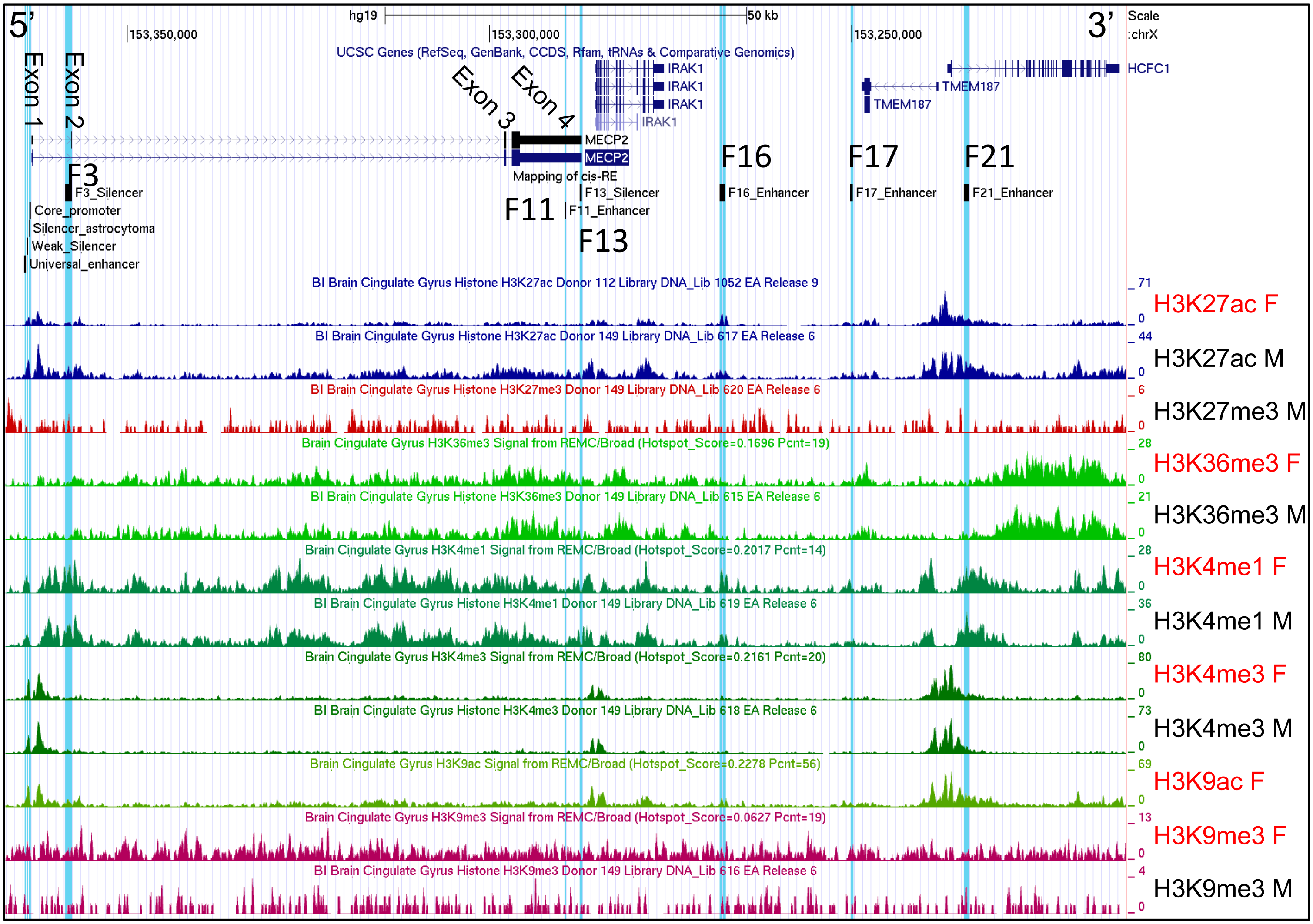


(B) Adult Mid Frontal lobe

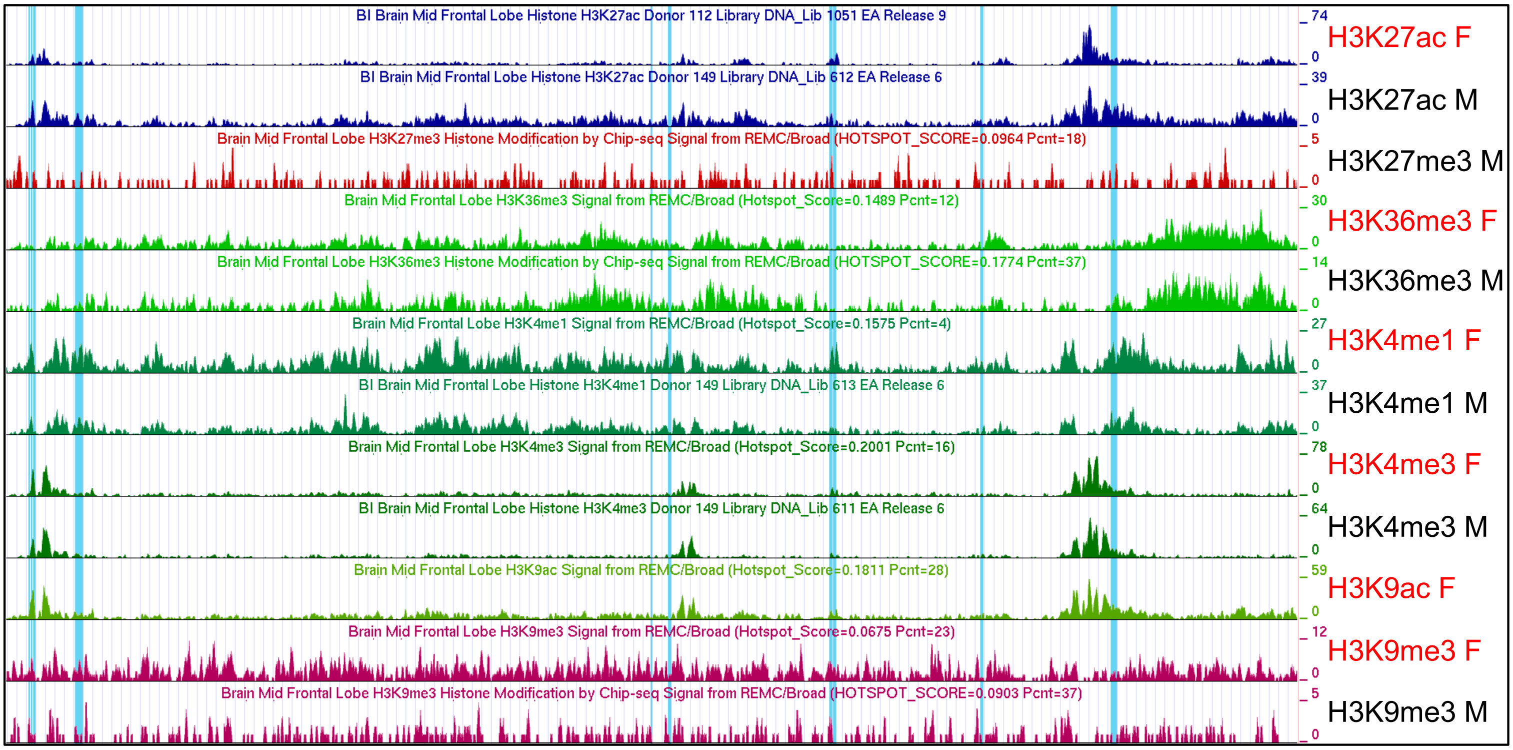

Supplementary Figure S2. (A) Adult Cingulate Gyrus, (B) Mid Frontal lobe, (C) Adult Anterior Caudate, (D) Adult angular gyrus, (E) Adult Substantia Nigra, and (F) Adult Inferior Temporal Lobe Histone PTMs of Males and Females.

(C) Adult Anterior Caudate

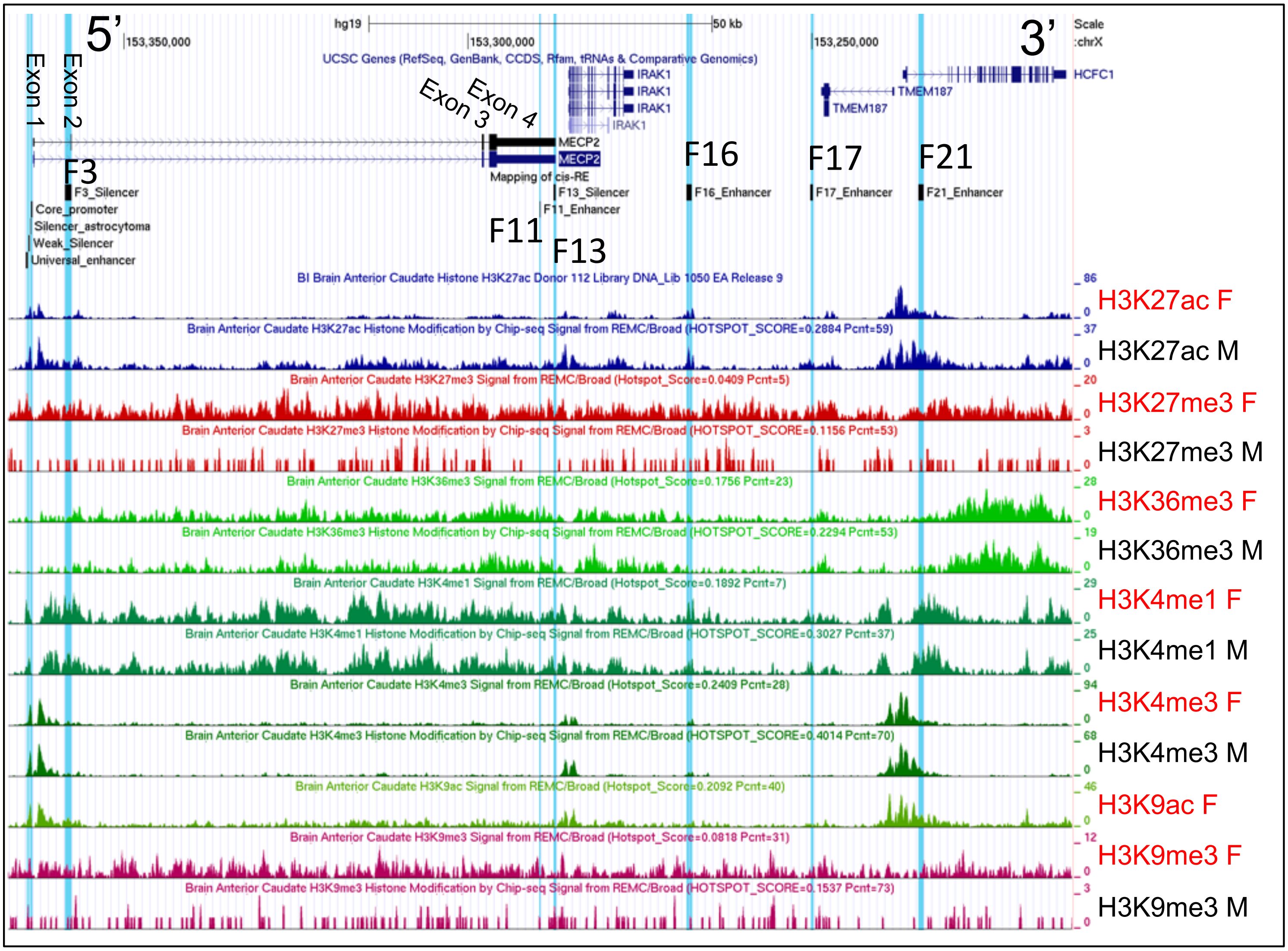


(D) Adult angular gyrus

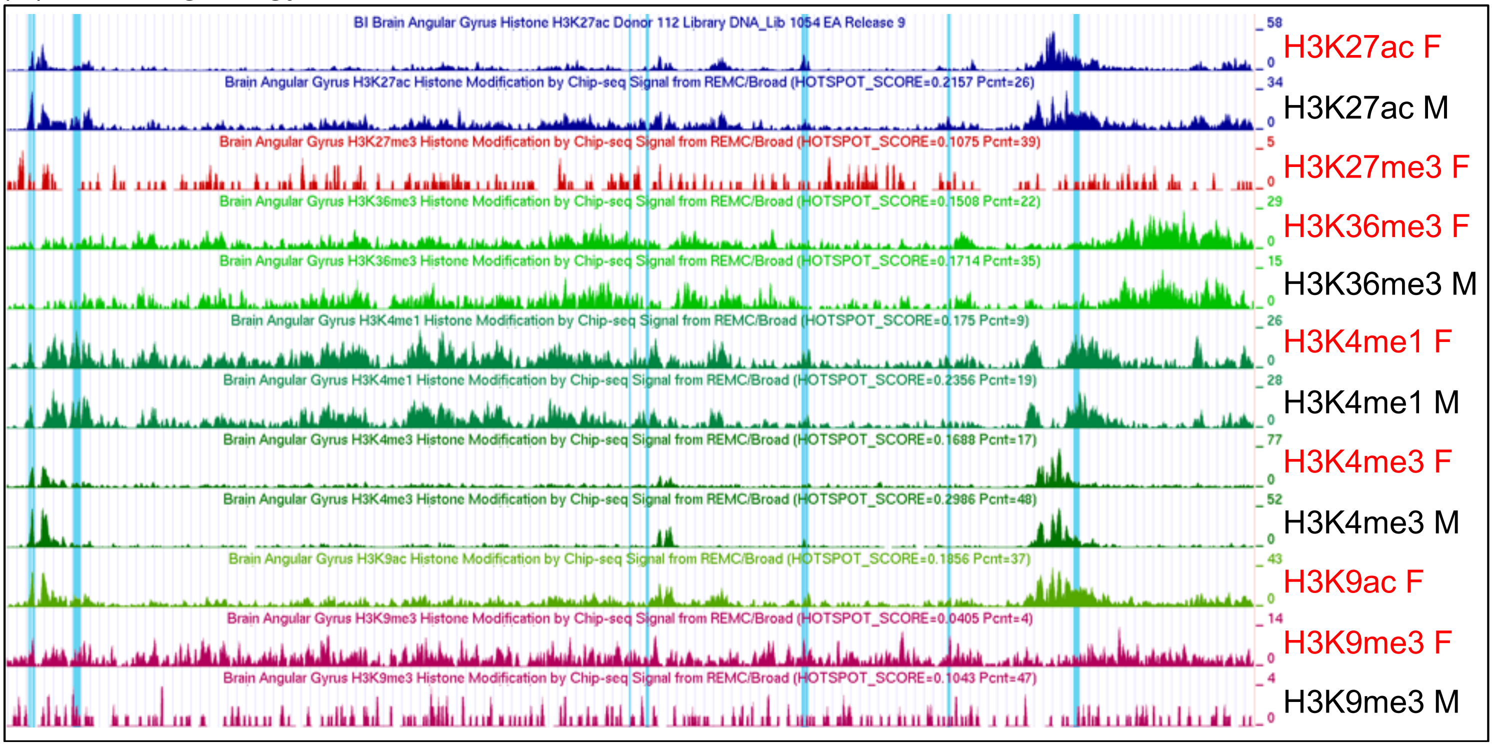

Supplementary Figure S2. (A) Adult Cingulate Gyrus, (B) Mid Frontal lobe, (C) Adult Anterior Caudate, (D) Adult angular gyrus, (E) Adult Substantia Nigra, and (F) Adult Inferior Temporal Lobe Histone PTMs of Males and Females.

(E) Adult Substantia Nigra

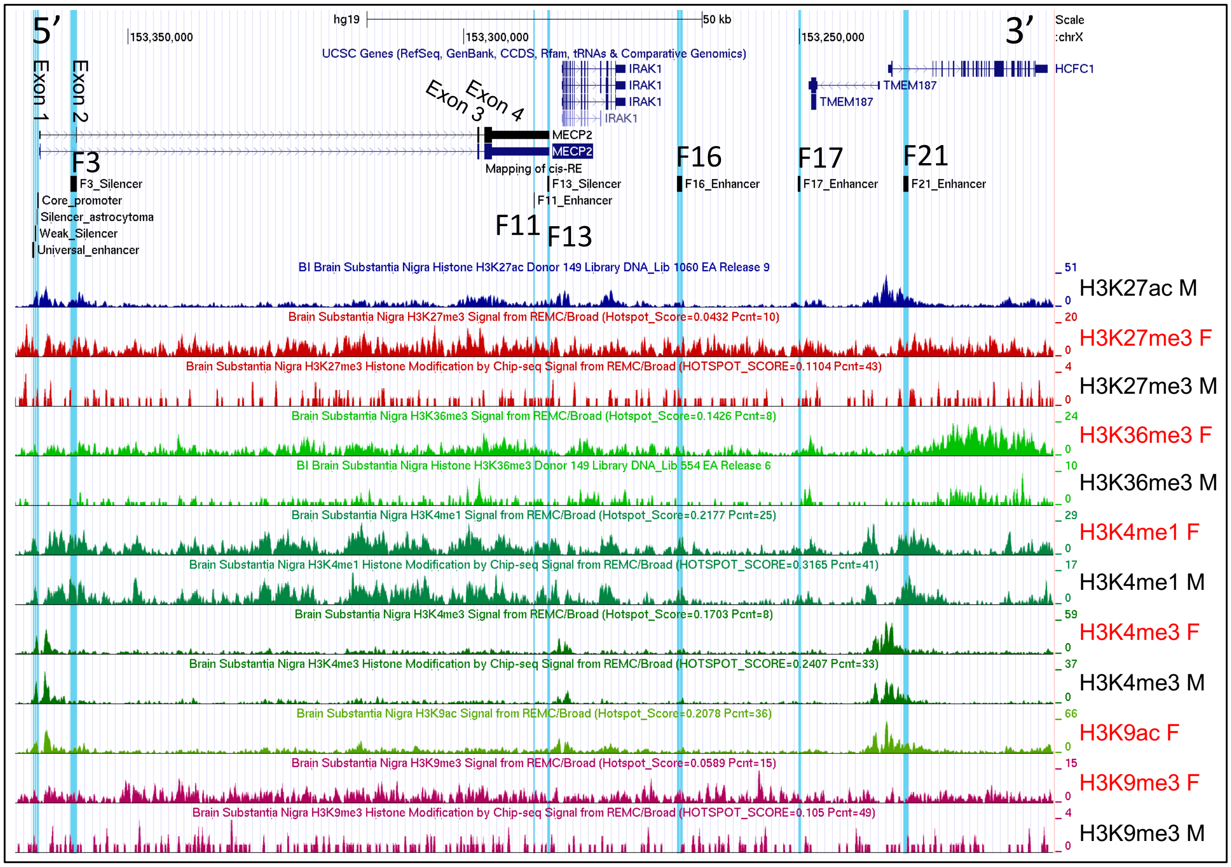


(F) Adult Inferior Temporal Lobe

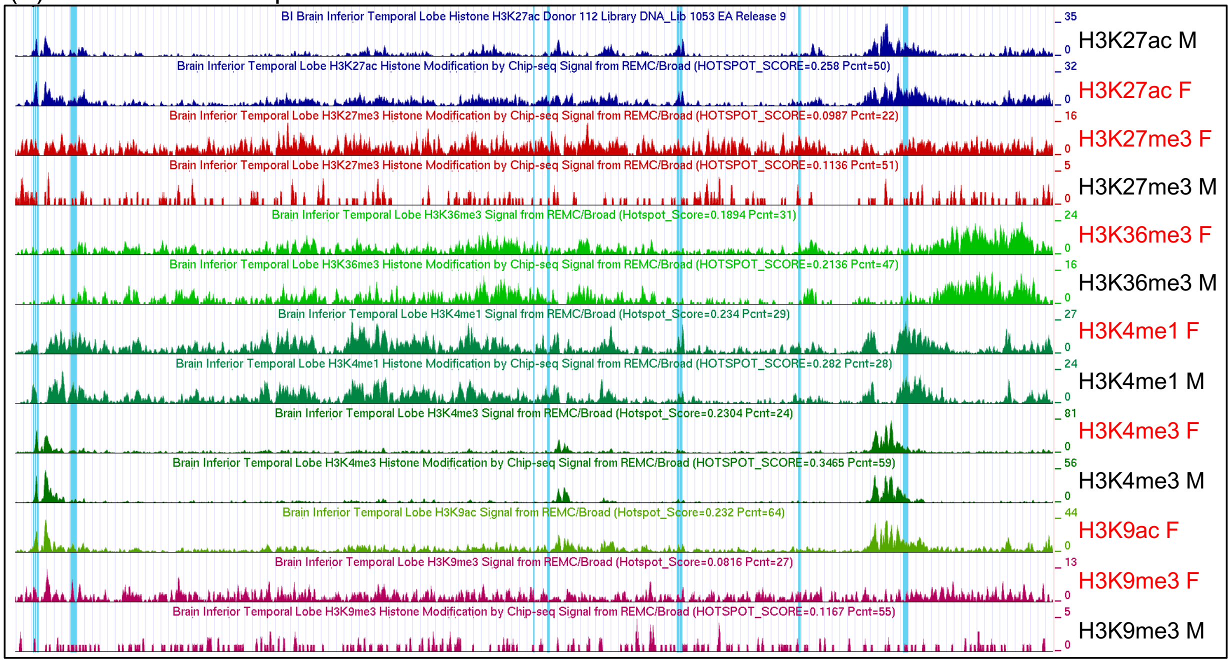


The area presented is the chrX:153,212,176-153,366,832 genomic region of the hg19 human genome assembly from the UCSC genome viewing browser. Direction the genetic sequence is 5′ – 3′ (sense orientation relative to the *MECP2* gene). Tracks displayed from the top to the bottom are: UCSC genes, mapping of cis-RE to genome, histone modification CHIP-seq data tracks. The original names of the data tracks are displayed on the top of each track. M: male, F: female. *Cis*-regulatory elements (REs) are highlighted blue in UCSC. The scale on the top represents a distance equivalent to 50 kb. *MECP2* exons are labeled on UCSC Genes data track. (A) Adult Cingulate Gyrus, (B) Mid Frontal lobe, (C) Adult Anterior Caudate, (D) Adult angular gyrus, (E) Adult Substantia Nigra, and (F) Adult Inferior Temporal Lobe. Histone PTM data is from GSE17312. The donors selected were: #112 (75 Y, female, disease-free, Rush University Medical Center), and #149 (81 Y, male, disease-free, Rush University Medical Center).

Supplementary Table S1. References for Figure 1.

| Feature | MeCP2E2 AA number | Reference |
|---|---|---|
| Methyl-CpG binding domain (MBD) | 78–162 | [65] |
| Transcriptional-repression domain (TRD) | 207–310 | [65] |
| High mobility group-like domain 1 (HMGD1) | 2–77 | [65] |
| Histone H3 methyltransferase interaction sequence (H3-M-T IS) | 78–102 | [65] |
| High mobility group-like domain 2 (HMGD2) | 163–206 | [65] |
| Nuclear localization signal 1 (NLS1) | 174–190 | [65] |
| Nuclear localization signal 2 (NLS2) | 253–269 | [65] |
| Alternative DNA binding domain (aDBD) | 198–305 | [65] |
| C-terminal domain α | 310–354 | [65] |
| C-terminal domain β | 355– 486 | [65] |
| Group 2 WW motif-containing protein interaction sequence (WW-2 IS) | 384–387 | [65] |
| AT hook 1 | 185-197 | [49] (P51608) |
| AT hook 2 | 265-277 | [49] (P51608) |
| TBL1XR1 interaction sequence (TBL1XR1 IS) | 285-309 | [49] (P51608) |
| NCoR/SMRT interaction domain (NID) | 269-309 | [22] |

# Supplementary Table S2. Links to the UCSC sessions.

| | |
|---|---|
| Figure 2 | https://genome.ucsc.edu/s/shevkopd/Roadmap%20Epigenome%20-%20Hippocampus%20-%20Histone%20CHIP-seq |
| Figure 3 | https://genome.ucsc.edu/s/shevkopd/Roadmap%20Epigenome%20-%20All%20brain%20regions%20-%20Histone%20CHIP-seq |
| Figure 4 | https://genome.ucsc.edu/s/shevkopd/Roadmap%20Epigenome%20-%20Fetal%20DNase |
| Figure 5 | https://genome.ucsc.edu/s/shevkopd/ATAC-seq%2025%20Brain%20Regions%20-%20GSE211822 |
| Figure 6 | https://genome.ucsc.edu/s/shevkopd/DNA%20methylation%20-%20gyrus |
| Figure 7 | https://genome.ucsc.edu/s/shevkopd/Summary%20-%20Segments |
| Supplementary Figure S1 | https://genome.ucsc.edu/s/shevkopd/hg38%20-%20BLAT%20of%20Ensembl%20cDNA |
| Supplementary Figure S2 | https://genome.ucsc.edu/s/shevkopd/Roadmap%20Epigenome%20-%20Substantia%20Niagra%20-%20Histone%20CHIP-seq<br>https://genome.ucsc.edu/s/shevkopd/Roadmap%20Epigenome%20-%20Temporal%20Inferior%20Lobe%20-%20Histone%20CHIP-seq<br>https://genome.ucsc.edu/s/shevkopd/Roadmap%20Epigenome%20-%20Anterior%20Caudate%20-%20Histone%20CHIP-seq<br>https://genome.ucsc.edu/s/shevkopd/Roadmap%20Epigenome%20-%20Angular%20Gyrus%20-%20Histone%20CHIP-seq<br>https://genome.ucsc.edu/s/shevkopd/Roadmap%20Epigenome%20-%20Gyrus%20-%20Histone%20CHIP-seq<br>https://genome.ucsc.edu/s/shevkopd/Roadmap%20Epigenome%20-%20MidFrontal%20-%20Histone%20CHIP-seq |
